# Speed and quality of complex strategic decisions

**DOI:** 10.1073/pnas.2531472123

**Published:** 2026-05-13

**Authors:** Uwe Sunde, Dainis Zegners, Anthony Strittmatter

**Affiliations:** ^a^ Economics Department, Ludwig-Maximilians-Universität München, München 80539, Germany; ^b^ Department for Technology and Operations Management, Rotterdam School of Management, Erasmus University, Rotterdam 3062 PA, Netherlands; ^c^ Faculty of Business and Economics, UniDistance Suisse, Brig-Glis 3900, Switzerland

**Keywords:** response times, uncertain evaluation, drift-diffusion model

## Abstract

Many strategic decisions involve both substantial complexity and time pressure, but the association between decision speed and decision quality of cognitively demanding strategic decisions is not well understood. This paper presents evidence on this question using a setting with exceptionally detailed and precise information about decision times and decision quality—it analyses move-by-move data from in-person professional chess tournaments. Decision quality is measured by comparing actual moves to a computational benchmark of best moves constructed using the artificial intelligence of a chess engine. The results show that faster decisions are associated with higher decision quality, even after accounting for computational complexity, distinctiveness between alternatives, and time pressure. Greater computational complexity and lower distinctiveness between move alternatives are associated with longer decision times, whereas greater time pressure is associated with shorter decision times. All three factors are associated with lower decision quality. We discuss the findings against the predictions of different decision models in which individuals sequentially acquire information about alternatives with uncertain valuations, extending theories originally developed in the context of nonstrategic decisions to a strategic environment.

## Significance

The association between the speed and quality of complex strategic decisions is a priori unclear. Taking more time to make a decision may result in a better thought-out decision of higher quality, but it may also reflect a greater perceived difficulty and hence be linked to lower decision quality. Empirical evidence on this question remains scarce and often relies on observations for different decision makers in contexts that are not strictly comparable. This paper contributes by documenting a robust negative association between the time spent on a decision and its quality, using move-by-move data from professional chess tournaments that allow for comparisons of decisions made by the same player against the same opponent under varying configurations.

Strategic decisions in many contexts involve both substantial complexity and time pressure. However, the association between decision times and decision quality is unclear, and evidence in the context of strategic decisions remains incomplete. Conceptually, the relation between the speed and the quality of decisions is a priori ambiguous because different mechanisms can work in opposite directions. Taking more time to make a decision may result in a more accurate and well-considered choice, implying that slower decisions should be of higher quality. Alternatively, taking more time may be associated with lower decision quality if the decision is genuinely more difficult for the decision maker: The greater the difficulty and perceived complexity of a decision, the longer the deliberation time and the lower the quality of choices. Such perceived complexity may stem from higher subjective difficulty or from limited information about the implications of different decision alternatives. In addition, fast decisions may indicate that the decision maker has a strong intuition about the right choice or relatively accurate information early in the decision-making process, so that the value of additional deliberation or information gathering is relatively low.

Here, we present an empirical investigation of the association between decision speed and decision quality for cognitively demanding decisions in a complex strategic decision environment with high stakes: professional chess. The investigation complements the mixed existing evidence on the association between the timing of decisions and their quality. This evidence is mainly based on experiments that relate to simple individual choices or stylized games, often in lab settings. Such settings enable controlled variation of certain aspects of the choice problem and facilitate the isolation and interpretation of specific mechanisms. In contrast, our setting involves strategic decisions that are more complex than those typically examined in experiments, identifying variation is not experimentally randomized, and the focus is limited to a highly select group of decision-makers. Nevertheless, chess is a conceptually interesting case for investigating the relationship between decision speed and quality in complex decisions by providing a high-stakes setting with extraordinarily rich and precise information about the context in which decisions are made. In particular, data from in-person games contain direct measures of the time to make a decision, move-by-move data involve variation in decision problems, and the reanalysis of data using chess engines delivers information about the computational complexity and difficulty of these decision problems. All these measures exhibit considerable variation within a game, which allows focusing on decisions of the same player in different configurations.

The decision-making process in chess can be viewed as solving a complex strategic decision problem in which the decision maker is uncertain about the quality of a particular move and, due to time restrictions, faces costs for gathering more information by cogitating over the various own decision alternatives and the respective strategic options of the opponent, when determining the optimal move. Decisions in chess are made sequentially, and the main goal of the player is the quality of the move, not the speed with which a decision is made. However, decision speed carries option value by impacting the time budget available for future moves, and it may also have strategic value by signaling dominance or weakness, or by inducing time pressure on the opponent. Ultimately, the objective remains to maximize the quality of play. This is conceptually very similar to the decision problems that decision makers often face in various other contexts. For this reason, professional chess represents a unique source of data of high-resolution and high accuracy on the behavior of decision makers in a competitive setting with high incentives, in which they are repeatedly observed while solving complex strategic choice problems under time pressure, and with varying degrees of difficulty and available time to make their decisions.

The empirical analysis is based on information about decision times and move quality for more than 215,000 moves from around 3,600 in-person over-the-board games between professional-level chess players. Decision time and decision quality are measured with high accuracy. Specifically, for each move, the time taken for the decision is precisely recorded, and the quality of the decision is assessed against the benchmark of the best possible move suggested by a cutting-edge chess engine’s artificial intelligence. This assessment involves comparing the player’s chosen move in a specific configuration of chess pieces on the board to the move recommended as optimal by the engine in the same configuration. This establishes a powerful metric for evaluating a move’s quality, drawing on the chess engine’s search algorithm that explores the game-tree arising from a given chess position under the game-theoretic assumption of best-move responses by the opponent. While this metric depends on the specific algorithmic choices implemented in the chess engine and is not perfect, the substantial gap in playing strength between modern engines and even the strongest human players makes this a highly informative benchmark for assessing decision quality that has been widely adopted by prior research e.g. refs. 1–4.

In addition, the use of chess data together with a chess engine makes it possible to measure three additional factors relevant to the decision problem. The first is time pressure, measured by the remaining time available, which constrains decision times and can affect decision quality. In the analysis, time pressure is measured by the remaining time a player has under different standardized time controls, each imposing different time constraints on players. The second factor is computational complexity of the decision problem. As a baseline measure, the analysis uses the number of nodes the chess engine needs to evaluate to reach a fixed search depth in a given configuration on the chess board. Alternative measures of complexity involve the search depth needed for identifying the best move, and whether a chess engine trained to replicate human-style play without searching the game-tree is able to determine the best move. The third factor is the difficulty of distinguishing between decision alternatives. This is measured by the evaluation gap between the best and the next-best move in a given board configuration, as determined by the chess engine. An alternative interpretation for the evaluation gap is that it provides a proxy for the value of reasoning to find the best move.

Analyzing the association between decision speed and decision quality on the basis of move-by-move data for many decisions of the same individual decision makers allows us to account for systematic heterogeneity across individual decision makers or across specific player pairings that may lead to particular playing styles. The analysis thus reveals evidence for the association between decision speed and decision quality while accounting for other patterns of decision-making or systematic influences that might affect the speed–quality relationship in between subject comparisons.

## Results

Table 1 presents the main results for the association between decision speed and the decision quality. The results are based on multiple regression analysis for three common time control formats of chess games: Classical chess, Rapid chess, and Blitz chess. These formats impose different overall time budgets for each player in a game and thereby create varying levels of time pressure, which may influence not only how quickly moves are made but also the strategic approach players adopt in their decisions. Classical chess generally provides the most generous time controls, with at least 2 h thinking time, allowing for deep calculation of moves. Blitz chess, by contrast, typically allows between 3 and 10 min per player, requires rapid decision-making, and involves more tactical play based on intuition and pattern recognition. Rapid chess usually allocates between 10 and 60 min per player, striking a balance between these two extremes. To focus on genuine decision-making, the analysis discards the first 15 moves for each player in all game types, since these opening moves are often played quickly and almost automatically from memory, providing little information on the decision process.

**Table 1.** Baseline results

	Dependent variable: Best move (Dummy)								
	Classical			Subset:Rapid			Blitz		
	(1)	(2)	(3)	(4)	(5)	(6)	(7)	(8)	(9)
*Decision time*									
Time spent on move (min.)	−0.023*** (0.0005)	−0.018*** (0.0005)	−0.021*** (0.0007)	−0.134*** (0.003)	−0.108*** (0.003)	−0.131*** (0.005)	−0.666*** (0.019)	−0.550*** (0.018)	−0.744*** (0.025)
*Time budget*									
Remaining time (min.)		0.0008*** (0.0002)	0.0006*** (0.0002)		0.006*** (0.002)	0.005*** (0.002)		0.022*** (0.008)	0.010 (0.008)
*Complexity*									
N Mega-nodes computed (log)		−0.029*** (0.004)	−0.034*** (0.004)		−0.052*** (0.005)	−0.054*** (0.005)		−0.067*** (0.004)	−0.069*** (0.004)
*Evaluation gap*									
Distance second best move (log)		0.197*** (0.004)	0.239*** (0.007)		0.174*** (0.005)	0.194*** (0.008)		0.152*** (0.005)	0.159*** (0.006)
*Interactions*									
Time spent × Remaining time			0.0003*** (0.00002)			0.010*** (0.0008)			0.312*** (0.022)
Time spent × N Mega-nodes			−0.004*** (0.0008)			−0.010 (0.008)			−0.069* (0.036)
Time spent × Dist. second best			0.021*** (0.002)			0.072*** (0.017)			0.190*** (0.056)
Move observations	80,633	80,633	80,633	62,825	62,825	62,825	73,945	73,945	73,945
Game observations	1,497	1,497	1,497	1,007	1,007	1,007	1,181	1,181	1,181
Player-game fixed effects	Yes	Yes	Yes	Yes	Yes	Yes	Yes	Yes	Yes
Control move number	Yes	Yes	Yes	Yes	Yes	Yes	Yes	Yes	Yes
Control evaluation position	Yes	Yes	Yes	Yes	Yes	Yes	Yes	Yes	Yes

*Note:* The table presents OLS estimates. Columns (1)–(3) only include games played with a classical time control; columns (4)–(6) only games with a rapid time control; and columns (7)–(9) games with a blitz time control. The evaluation of the current position is controlled for using two dummy variables indicating whether the player to move is in a favorable (>0.5 pawn units) or unfavorable (< −0.5 pawn units) position. All independent variables have been demeaned, allowing main effects to be interpreted as marginal effects at the mean when interaction terms are present. SEs are clustered at the game level, and significance levels are indicated as follows: *: *P* < 0.1, **: *P* < 0.05, and ***: *P* < 0.01.

The dependent variable is move quality, measured by a binary indicator whether the best move—according to the chess engine’s evaluation—is played, or not. The multiple regression models condition on a rich set of control variables to account for systematic heterogeneity that might affect the coefficient of decision time. All specifications include controls for (interacted) player-game fixed effects to account for systematic variation in playing style or other factors that influence players’ decisions in a given game on a given day, fixed effects for the number of moves previously played in a game to account for variation along the course of the game and fatigue, and a control for the evaluation of the current position on the board by the chess engine to account for systematic differences in offensive or defensive positions.

Column (1) of Table 1 documents a significantly negative association between the time spent on a given decision (move) and the decision quality in classical games of chess. This implies that faster decisions are associated with higher quality of the decision in terms of the likelihood of playing the best move according to the chess engine. A possible interpretation is that longer deliberation often reflects greater difficulty or uncertainty, which lowers decision quality, whereas fast decisions may signal strong intuition or quick recognition of the correct move.

In Column (2), we additionally control for time budget (time remaining on the clock), computational complexity (number of nodes computed by the chess engine), and the distinctness of decision alternatives (evaluation gap between the best and next-best move). The association between decision time and decision quality remains significantly negative. A larger time budget is associated with higher decision quality, greater computational complexity with lower quality, and more distinct alternatives with higher quality. In other words, decisions are better when players have more time, when the problem is less complex, and when the best move is easier to distinguish from the next-best.

The findings for time budget are consistent with the hypothesis that, conditional on decision time, a larger time budget is associated with higher decision quality, possibly because of reduced stress or cognitive load. This effect is unlikely to reflect physiological factors such as fatigue, which are accounted for by controlling for the number of moves played.

Although time budget, computational complexity, and distinctness are only weakly correlated with each other, all three are strongly correlated with decision times. Players spend more time when they have a larger budget, when problems are more complex, and when alternatives are harder to distinguish (*SI Appendix*, Table A4).

Column (3) of Table 1 reports results from a specification that adds interaction terms with time budget, computational complexity, and distinctness. The main coefficient of the speed–quality association remains significantly negative, indicating that faster decisions are consistently associated with higher quality even after accounting for these interactions. The other coefficients are also qualitatively and quantitatively similar to those in Column (2), but the significant interactions show that the speed–quality association varies with these factors. Relative to the overall negative association, longer decision times are associated with comparatively higher decision quality when time pressure is lower, when computational complexity is lower, and when the best move is more distinct from the next-best. By contrast, the negative association is more pronounced under high time pressure, greater computational complexity, or smaller evaluation gaps, when the best move is harder to distinguish. It may seem counterintuitive that longer deliberation is not associated with higher decision quality in complex or ambiguous situations, but this aligns with the notion that greater complexity prolongs deliberation without improving decisions and that intuition and experience can play a central role.

Columns (4)–(6) of Table 1 show a similar pattern for Rapid chess, and Columns (7)–(9) report the same for Blitz chess. Across all specifications, the association between decision time and decision quality remains negative. The coefficients increase in magnitude, which indicates that the negative association between decision time and decision quality is stronger under the tighter time controls of Rapid and Blitz chess.

The coefficient of the time budget is larger in magnitude for Blitz chess. However, the SEs also increase, as Blitz games offer little variation in time budget over the course of a game, so the observed differences may simply reflect noise. The coefficient of computational complexity is also larger in magnitude for Rapid and Blitz, suggesting that complexity may exhibit a more negative association with decision quality under fast time controls. In contrast, the coefficient of the evaluation gap is smaller in magnitude for Rapid and Blitz, suggesting that distinctness is less positively associated with decision quality when time is scarce.

Columns (6) and (9) of Table 1 show that the interaction terms between time spent and time budget, computational complexity, and evaluation gap become larger in magnitude for Rapid and Blitz chess. This suggests that, compared to Classical chess, the negative association between decision time and decision quality is more pronounced when time pressure is high, positions are complex, or alternatives are difficult to distinguish. A possible explanation is that in Rapid and Blitz the severe time constraints encourage a strategy based primarily on intuition and heuristics rather than on systematic calculation. As a result, the effects of time pressure, complexity, and evaluation gap on decision quality become more visible, which is reflected in the larger interaction terms.

One of the main findings in Table 1 is that the negative association between decision time and decision quality is quantitatively much larger in games with shorter time budgets (Rapid and Blitz). This is not surprising given the differences in time control, since the effect of spending an additional minute on a move should vary across formats.

**Fig. 1.**
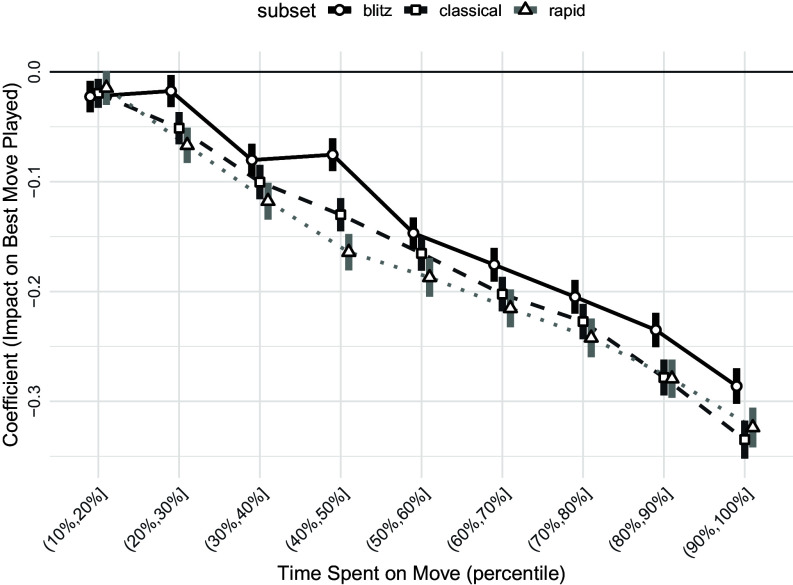
Decision time and move quality across the time distribution. *Note:* The figure presents estimates from a flexible model. Decision times are divided into ten equally sized bins (by number of observations), defined separately for Classical, Rapid, and Blitz games. All coefficients are expressed relative to the 10% quickest moves (the [0, 10%] bin). For example, the coefficient for the bin (70%, 80%] shows the probability of playing the best move for that bin relative to the fastest 10% of moves, within each time control format. The regression specification is otherwise the same as in Table 1, including controls for available time budget, computational complexity, and distinctiveness, as well as player–game fixed effects, move-number fixed effects, and indicators for favorable or unfavorable positions. Error bars show 95% CIs with SEs clustered at the game level.

Fig. 1 presents estimates from a model designed to provide a better comparability of the estimated coefficients across game types. Instead of measuring the effect of an additional minute spent on a move, which has different implications in Classical, Rapid, and Blitz games, decision times are divided into ten equally sized bins (by number of observations), defined separately for each time control format. By benchmarking decision speeds relative to each format, the results become more comparable across Classical, Rapid, and Blitz games.

The figure shows that the association between decision time and decision quality is monotonically declining and approximately linear across the entire distribution.^*^ This suggests that the negative association between time spent on a decision and its quality is not driven by extreme cases, such as very fast obvious moves. Moreover, once decision times are grouped into frequency bins, the estimates indicate that the associations are quantitatively comparable across Classical, Rapid, and Blitz games. Further analyses, not reported in the tables, suggest that the same pattern holds when time spent on a move is normalized by the regular time budget or standardized within the sample.

In sum, the results document a significant negative association between the time taken to make a strategic decision and the quality of the decision. This association appears across different commonly used time control formats with varying time budgets and persists after controlling for time pressure, computational complexity, and the difficulty to distinguish between decision alternatives. A possible interpretation is that longer decision times reflect greater subjective difficulty experienced by the player when evaluating the move. This perceived difficulty is conceptually distinct from time pressure (captured by remaining time), from computational complexity (the number of nodes the chess engine must search to reach a given depth), and from the discriminability of the best move (the evaluation gap between the best and second-best move), all of which are controlled for in the respective regression models.

### Additional Results.

The negative association between decision times and decision quality can be explained by longer decision times reflecting a greater perceived complexity or difficulty of the decision. The question of how to measure complexity has been an active area of research.

The computational complexity of a decision, which is held constant in the previous analysis, is a widely used proxy of decision difficulty. It reflects the computational resources required to identify the best move. Yet, the finding of a negative association between time spent and decision quality suggests that even when controlling for the measure of computational complexity, some decisions involve more deliberation but nonetheless have poorer outcomes. A possible explanation is that the specific proxy measure of computational complexity, in terms of node counts, may not adequately capture the relevant dimension of the computational difficulty associated with finding the optimal decision. Alternatively, measures of computational complexity might not fully capture the complexity of a decision as subjectively perceived by the individual decision maker in the specific decision context, which is closely related to the cognitive costs of identifying the best move, but nevertheless distinct.

To address this question and to ensure that the results are not driven by specific modeling and measurement choices, a series of robustness analyses based on alternative specifications and complementary measures was conducted.

First, we replicated the analysis for alternative measures of computational complexity that capture different aspects of decision difficulty. These include the search depth at which the best move is first identified by the chess engine, the number of legal moves available in a given position, and the absolute change in the evaluation between shallow and deep search. Second, we incorporate measures derived from a chess engine specifically trained to mimic human play (“Maia”). This engine uses a neural network trained on human games to predict players’ moves based on the current position, without searching the game tree. It therefore approximates moves based on pattern recognition and intuition rather than time-intensive calculation of optimal sequences. The use of this chess engine constitutes an extension to the literature as such an engine trained to replicate human behavior might provide a better approximation to the subjective difficulty experienced by players. Discrepancies between evaluations of a computational engine based on traditional search and a human-trained engine might be indicative of a role of subjective (as opposed to purely computational) complexity. Specifically, the analysis examines: 1) whether Maia’s suggested move matches the actual move played, 2) whether it matches the computationally best move, and 3) how closely Maia’s evaluation aligns with the optimal evaluation.

The first set of results, reported in *SI Appendix*, Table A3 for Classical games, confirm the robustness of the main findings for alternative measures of complexity. The negative association between decision time and decision quality remains strong and quantitatively similar across specifications with different measures. The alternative complexity measures also show a nega- tive association with decision quality, and the inclusion of these alternative measures does not alter the magnitude of the association between time spent and quality. Interestingly, the findings document that for positions in which the best move suggested by the human-trained engine is closer to the computationally optimal move, the decision quality is systematically higher. This supports the interpretation that the human-trained prediction measures capture the subjectively perceived difficulty of a decision, with decision quality being higher when the best move is easier for humans to identify. Across all specifications and for all complexity measures considered, higher complexity is associated with longer deliberation times (*SI Appendix*, Tables A4 and A5.) This further supports the interpretation that longer decision times reflect greater subjectively perceived difficulty.

The second set of results replicates the main analysis using a different benchmark for measuring decision quality. Building on the previous discussion, quality is not measured by whether a move coincides with the computationally best choice identified by a chess engine that is based on a traditional tree search algorithm. Instead, decision quality is evaluated by comparing the actual move to the prediction of a chess engine trained to approximate a human style of play (“Maia”). This alternative benchmark may be closer to the types of decisions that players are likely to consider, which are often based on intuition and experience rather than deep calculation, or which might be constrained by cognitive limitations or impacted by human-specific strategic considerations.

The estimation results for this alternative measure of decision quality as dependent variable deliver a pattern very similar to that in the baseline analysis (*SI Appendix*, Table A6 and Fig. A1). In particular, the association between time spent on a move and the likelihood of selecting the best move predicted by the engine remains significantly negative. The coefficient estimate is quantitatively even larger than in the baseline specification with the likelihood of selecting the computationally best move as the dependent variable (see *SI Appendix*, Table A6 compared to Table 1). A similar pattern holds when comparing subsamples in which the computationally best move coincides with the predicted human move to those in which it does not (*SI Appendix*, Table A7). In both cases, the association remains significantly negative, but it is more pronounced when the computationally best move also aligns with the prediction of a human-like chess engine. This is an indication that deviations between computationally best and human-like benchmarks are not the main driver of our results. The results are also robust when decision quality is measured at the intensive margin, defined as the difference in pawn points (log-modulus) between the computationally best move and the move actually played (*SI Appendix*, Table A8), or when quality is evaluated using best moves determined by a computational engine with limited search depth (*SI Appendix*, Table A9).

Third, additional analyses examining the role of time as a potential strategic tool to influence the opponent’s future moves do not alter the main findings. Including controls for move quality and decision times in previous moves, in order to account for potential correlations and long-term strategies within games, does not materially affect the estimated associations between decision times and decision quality (*SI Appendix*, Tables A10 and A11).

Fourth, “surprise” moves by an opponent might affect a player’s decision time and quality. While measuring such “surprise” moves is conceptually difficult, several indicators of the opponent’s previous move (best possible move, machine-predicted move, or different measures of opponent’s previous move quality) can be used as proxy measures. The so-measured surprise moves by the opponent indeed increase the time spent on a player’s subsequent move (*SI Appendix*, Table A12), but their inclusion does not change the main findings regarding the negative association between decision time and decision quality (*SI Appendix*, Table A13). Likewise, the negative association is robust to excluding positions in which the previous move involved a capture by the opponent or created a check (*SI Appendix*, Table A14). Both types of events could introduce sudden changes in the game, yet the results show that excluding them does not alter the findings, indicating that the observed relationship is not driven by positions affected by unexpected shifts in play.

Fifth, additional robustness checks reveal a similar pattern of results across different phases of the game and player standings. The association between time spent on a move and move quality remains negative when decisions are split by game phase (middle game versus endgame, *SI Appendix*, Table A15), or by the overall number of moves in a game (*SI Appendix*, Table A16). Likewise, the negative association holds regardless of whether players are in favorable positions (with higher winning chances than the opponent) or unfavorable ones (with lower winning chances) at the time of decision-making (*SI Appendix*, Table A17).

Sixth, we find no evidence that the negative association between decision time and decision quality varies systematically with player strength (*SI Appendix*, Table A18).

## Discussion

The result of a robust negative relationship between the time taken to make a strategic decision and the quality of the decision contributes to a recent literature on the relation between endogenous decision times and decision performance, and on the underlying processes of reasoning in strategic decision-making contexts (e.g., 5, for a survey). Existing studies of response times are mostly based on notions of two-system models that distinguish between instinctive and cognitive (or contemplative) decision-making (6, 7), and that classify individuals into instinctive and contemplative types (8, 9). While this work typically focuses on observations of the behavior of the same individual in different games, our data comprise many observations of decisions made by the same individual in the same decision context and within the same pairing with a particular opponent, but with varying configurations that differ in complexity and time pressure. This implies that the observed patterns emerge within a more controlled decision environment and relate to the parameters of the decision process rather than different types of decision makers or decision contexts.

Few studies have used repeated strategic interactions in the lab to measure the strategic complexity of a decision. For example, recent work finds a negative relationship between response time and performance in a *p*-beauty contest with feedback (10), which suggests that decision makers may find certain decisions more difficult than others. Our result of a negative relation between decision time and decision quality is consistent with these findings, but our results are based on within-subject comparisons using various measures of decision complexity and time pressure. In particular, our analysis accounts for the difficulty of a decision problem as reflected by the computational complexity of finding the best move and by the evaluation gap between decision alternatives based on the artificial intelligence of a chess engine, whereas previous literature has used potentially endogenous measures based on the time participants spend on a decision problem (8, 10). By exploiting within-subject variation, our analysis also holds constant beliefs about the opponent’s depth of reasoning, playing style, and behavioral peculiarities, thus focusing attention on cognitive limitations and perceived difficulty as the main determinant of decisions. In addition, by explicitly accounting for variation in time pressure, computational complexity, and the evaluation gap (which can be a proxy for both, discriminability of optimal choices in the sense of lower complexity or for a greater value of reasoning by indicating more costly mistakes with a larger gap), our analysis also addresses concerns about these as potential confounders in the respective literature (see, e.g., refs. 5 and 11, for a discussion of this point and the need for accounting for decision difficulty). The distinct influence of response time, remaining time, and computational difficulty (in terms of complexity and the evaluation gap) supports concerns about subjectively perceived choice complexity as a potential confounder in the relation between response times and performance (see refs. 5 and 11).

Our findings also contribute to an established literature in psychology and cognitive science that has studied the roles of analytical (slow) thinking as opposed to (fast) intuitive, pattern-oriented thinking for solving complex problems. Based on experiments with professional chess players, the main finding of this literature has been that pattern recognition constitutes a central aspect of the superior performance of experts, implying a link between fast decisions and high performance (12–14). Despite being questioned subsequently on the grounds of evidence that time pressure benefits stronger players who also have an advantage in analytical and intuitive thinking (15), this finding has been successfully replicated (16, 17). Our results complement the findings of this line of research by documenting a negative relation between decision times and decision quality, based on variation within the same player or player pair instead of between-player variation. Recent work using online chess has documented that decision times increase in the benefit of making the optimal move, as measured by the evaluation gap between the move suggested by a similar chess engine as used here compared to the move that the engine with search depth zero, suggesting that decision times proxy the value of computation (18). Related evidence has shown that time pressure affects decision times and the quality of selected moves, particularly in configurations when the value of additional computation is greater (19), that players adopt less risky moves under time pressure (20), and that lower-skilled players are more susceptible to overthinking (21). Our finding of a negative relation between decision times and decision quality, which holds conditional on time pressure and the value of computation as proxied by the evaluation gap, complement the findings of this line of research and suggest a pattern in the decision-making processes that determine decision times and quality simultaneously. This conclusion is further supported by the close similarity of the findings for three different time control formats with different time budgets and correspondingly different weight on analytical computation relative to intuitive pattern recognition in the process of decision-making.

The results also shed light on the underlying decision-making process. In particular, the negative relation between the decision time and decision quality after controlling for measures of computational (objective) complexity and time pressure is inconsistent with simple two-system models of decision-making, which predict that faster decisions, e.g., due to greater time pressure and lower subjective importance, are less likely to be based on deep reasoning and thus exhibit lower quality in complex settings (see, e.g., refs. 22 and 23). Instead, the negative correlation is consistent with two-system models in which decision speed and quality depend on the alignment of solutions based on an intuitive system of decision-making (which may be based on past experience) and cogitation (24–27). Earlier work has predicted that a larger time budget leads to better decisions by allowing for the processing of more decision alternatives see, e.g., ref. 28. Our findings are consistent with this prediction and confirm findings that additional time available for deliberation and a lower complexity of decision alternatives are associated with better performance (see, e.g., refs. 4, 19, and 29). However, our results also indicate the need to take decision times into account when analyzing the role of time pressure on decision quality, since the positive relation between remaining time and decision quality only holds when conditioning on decision time. This is consistent with findings that the observed distribution of choices and their correlates depend critically on controlling for response time (30). Our results disentangle the roles of the available time budget (which reflects the implicit cost of later decisions), and the complexity and discriminability of the decision (the latter being related to the uncertainty associated with a choice being optimal), on decision speed and quality.

The analysis of complexity of decisions relates to a recent strand of literature that has focused on how complexity affects behavior. Conceptually, complexity describes the difficulty of solving a decision problem or making a correct decision. A useful operationalization links complexity to the cost of identifying and implementing a procedure that transforms the primitives of the problem into a decision (31). In the present context, the procedure is the consideration that maps a particular position into a specific move. This conceptualization implies that the optimal procedure (the optimal strategy that entails a particular decision or move) does not necessarily coincide with the procedure that is chosen optimally under the constraint of complexity, that is, the consideration that maximizes the rewards of the move net of the cost to identify the respective consideration. The baseline measure of complexity implemented in the empirical analysis closely reflects the complexity associated with computational cost for evaluating a position, paralleling similar counts of choice alternatives that have been used in the literature (see, e.g. 32, 33, in the context of decisions under risk).^†^ The alternative measures of complexity capture related, yet slightly different, aspects, including the distinction between a purely computational measure and a measure that is designed to reflect human play based on pattern recognition rather than tree search. After conditioning on these computational measures of (task) complexity, the negative association between decision time and decision quality indicates the existence of unobserved heterogeneity across moves that correlates with longer decisions and lower quality. This heterogeneity is potentially best described as subjectively perceived (procedural) complexity (following the terminology of 31) and reflects cognitive uncertainty (as in, e.g., 35).

The joint determination of decision times and decision quality can be analyzed through the lens of different theoretical models. In the context of models of endogenous depth of reasoning (e.g., refs. 36 and 37), the optimal choice is the result of a trade-off between costs and benefits of additional reasoning, which is related to the number of cognitive steps or the degree of effort spent on information acquisition to reach a decision. The empirical results shown here, however, do not reflect this tradeoff, which applies for problems of the same cognitive equivalence class, i.e., problems that are equally difficult and that require the same form of reasoning. When decision makers are confronted with problems of varying difficulty due to the variation across configurations within the same game, the cost and path of reasoning vary, and hence a higher difficulty may lower the quality of the decision. Moreover, within this model class, the precise connection between decision quality and decision time depends on the mapping between steps of reasoning and decision time across the difficulty of problems, which would require imposing additional structure to obtain sharp predictions (see also 38). The estimated association between decision time and quality can be explained by decision makers being confronted with problems of varying difficulty due to the variation across configurations within the same game. A higher difficulty lowers the quality of the decision for any level of cognitive effort due to slower reasoning or smaller information gains from exerting effort. As a consequence, more difficult decisions can be associated with more time spent on the decision *and* lower quality. Importantly, this result is found even when conditioning on computational complexity of a decision, which suggests that the driving force behind the results is variation in the subjectively perceived (but unobserved) difficulty of a decision. Also, by focusing on within-game variation, our setting holds cognitive limitations of decision makers and their opponents fixed and controls for the value of reasoning, complementing the setting studied in some models of endogenous stochastic depth of reasoning (39).

A similar intuition emerges from models of optimal stopping and drift-diffusion see, e.g., ref. 40. These models have mainly been used to model decisions in simple nonstrategic decision problems, and existing empirical evidence is mostly restricted to lab experiments see, e.g., refs. 41–46. The finding of a negative association between decision time and decision quality is inconsistent with the prediction of standard drift-diffusion models, namely that longer decision times improve choice quality, conditional on the complexity or difficulty of the decision problem. The empirical results are consistent with the predictions of extensions of drift-diffusion models to settings in which the relative evaluations of decision alternatives are uncertain ex-ante. In such settings, the positive association between decision speed and decision quality dominates (see 47, 48, for details). Specifically, the decision of choosing the optimal move for a given configuration on the chess board can be viewed as the situation considered by drift-diffusion models with uncertain evaluations. Information acquisition occurs in the form of cogitation, which can be conceptualized as a diffusion process with drift and random stimuli, and a decision about a move is made when the cogitation process reaches a decision boundary.^‡^ As a consequence, the observation that a move is made is associated with a realization of decision time and decision quality, which is measured as the probability of making the best possible move in a given configuration conditional on making the decision at a certain time.^§^ Uncertainty enters into the evaluation of the choice alternatives. The decision maker has a prior belief about the evaluations, but is uncertain about the best move, either due to the analytical complexity of the problem or due to the strategic uncertainty. Hence, the decision maker incurs costs over time while receiving (independent) signals about the true evaluation of a move (which also have the form of drift plus random stimuli that follow a Brownian motion). Since the decision depends on the relative evaluation of the choice alternatives, the evaluation difference is a sufficient statistic for the decision. The larger the perceived evaluation difference, the more informative the signal (the larger the drift toward the boundaries). While the decision boundaries vary with decision time in a potentially nonmonotonic way, decision quality has been shown to decrease with decision time when aggregating many decisions of a decision maker who on average has correct priors about the difference in the evaluation of the respective choice alternatives (see refs. 47 and 48, for details). The probability of making the best possible move and the associated decision time are then affected by three mechanisms. The first is related to cogitation and information acquisition and implies that stopping later to make a move is associated with more information, which tends to increase the probability of choosing the optimal move. Two selection effects work in the opposite direction, as optimal decisions are associated with earlier endogenous stopping when signals are strong and thus more informative (i.e., they exhibit a larger drift and/or a less noisy stimulus process), or endogenous stopping occurs earlier when subjects experience a faster understanding of the optimal behavior (which is equivalent to a more discriminative prior and thus more informative signals). Importantly, signal strength and speed of understanding are related to subjective perceptions of the decision maker and may vary at the level of moves, leading to the observed negative association after controlling for time budget, computational complexity, and the evaluation gap. Viewed from this perspective, longer decision times reflect greater subjective complexity or difficulty associated with a particular decision, conditional on its computational complexity, which has an independent effect on longer decision times. Recent work on optimal stopping has pointed at the possibility of a nonmonotonic relationship between complexity and decision time even when the relationship between choice complexity and decision quality is monotonic (51). Our results do not indicate a nonmonotonic relation between decision time and decision quality or computational complexity, but the fact that perceived difficulty is unobserved makes this prediction difficult to test with our data. Nevertheless, our results suggest that the predictions of drift-diffusion models with uncertain evaluations of decision alternatives, which have mainly been used to model decisions in simple nonstrategic decision problems, are consistent with behavioral patterns in complex strategic decision contexts.

In line with this interpretation, our additional findings for effect heterogeneity show that spending more time on a decision exacerbates the negative associations of greater complexity, while spending more time on a decision when uncertainty about the best decision is lower is associated with relatively higher decision quality. These findings may be indicative of additional behavioral aspects that determine decision-making, as reflected in more elaborate models, such as dual-process diffusion models that account for alignment or conflict in inference about optimal decisions from a heuristic model of decision-making and a maximizing model of decision-making (see, e.g., refs. 26 and 52). The analysis here focused on the overall patterns of the relation between speed and decision quality in the context of endogenous timing of decisions, and abstracted from the analysis of systematic heterogeneity across individuals or the implications of strategic interactions. For instance, to the extent that cognitive processes involved in decision-making are affected by physiological factors, age might play a crucial role in cognitive performance see, e.g., ref. 2. Similarly, experience might interfere systematically with the way complexity or uncertainty shape decisions. A natural next step in the research agenda is to apply the methodology developed here to investigate the influence of individual-specific factors on behavioral mechanisms in more detail. Disentangling the role of strategic interactions in shaping behavior constitutes another interesting direction for future research.

## Materials and Methods

### Data and Measurement.

#### Data.

The analysis is based on detailed information about more than 215,000 moves from around 3,600 games of professional chess championships with substantial prize funds. The data cover games from tournaments with different time controls, classified as Classical, Rapid, and Blitz. The data contain more than 80,000 moves from almost 1,500 games of Classical chess games played in 37 tournaments during the years 2014–2017. All Classical games were played under regular time controls, which give each player a time budget of a minimum of 2 h thinking time to conclude the game.^¶^ In addition, the data include more than 62,000 moves from more than 1,000 games of Rapid chess championships and almost 74,000 moves from almost 1,200 games of Blitz chess championships in the years 2015–2016. Rapid chess games involve time controls of more than 10 but less than 60 min (typically 15 min with a 10-s increment per move), while Blitz chess involves time controls of more than 3 but less than 10 min (typically 3 min per player with a 2-s increment per move) per player.^#^ Analyzing games of chess with different time controls allows investigating behavior under different time budgets and, correspondingly, different types of play. Whereas Classical chess games allow players to strategically plan and calculate moves intensively, Rapid chess games require players to balance strategic calculation and tactical speed, whereas Blitz chess requires quick decision-making and tactical play. The data have been collected from an internet platform that broadcasted all professional over-the-board (in person) chess tournaments (www.chess24.com).^‖^ These tournaments include events such as national championships, qualifications tournaments for the World Championship, and the most prestigious invitational tournaments in the chess calendar. *SI Appendix*, Table A1 provides a list of the tournaments included in the dataset. Tournaments that are not officially organized by FIDE use slight variations of the official FIDE time control regime. The data contain detailed information about the players, including their past performance statistics in terms of their ELO rating.^**^ We restrict our analyses to games between professional players with an ELO rating of at least 2,500 at the time of the game.^††^

The data include precise information about the time consumed for each move and the remaining time budget. In addition, the move-by-move data comprise information about the exact configuration of the pieces on the board. We use this information to compute an evaluation of this configuration in terms of the relative standing of each player, the complexity of the configuration, and an evaluation of the move quality, using a chess engine as explained in more detail below. We exclude the first fifteen moves of each player in a game from all our analyses. These moves are typically the result of routine openings, which are intensively studied and memorized by players in preparation for a game.

#### Measuring decision quality.

To construct a measure of decision quality, we use a chess engine that computes the best possible move for a given configuration of pieces on the chessboard. In particular, we use the open-source chess engine STOCKFISH 17, which is considered to be one of the best programs available and has an estimated ELO rating more than 3,600 points.^‡‡^ In comparison, the currently highest rated human player had an ELO rating of 2,839 points in August 2025 according to the official rating list of the International Chess Federation FIDE, the current World Champion Gukesh Dommaraju, has an ELO rating of 2,752 points.^§§^

For each configuration, the chess engine searches the game-tree of all possible moves of white and black for a prespecified depth of *n* moves ahead, the so-called search depth. We restrict the engine to a search depth of *n* = 22 moves to save computational costs. The configurations at the respective end-nodes are evaluated by the engine using an evaluation function that considers aspects such as pieces left on the board, safety of the king, mobility of pieces, pawn-structure, etc. Based on this evaluation, the engine then searches for the best move using an algorithm conceptually similar to backward induction under the assumption of mutually best responses.^¶¶^

Concretely, the chess engine provides a measure of relative standing for a given configuration of pieces on the board. This measure reflects an evaluation of a player’s current position and is a proxy for the winning odds. The evaluation is measured in terms of so-called pawn units, where one unit approximates an advantage that is equivalent to possessing one more pawn.^##^ Based on this information we compute a measure of decision quality, in terms of the quality of play of a given player in a given configuration of pieces on the board. This measure is based on a comparison of the actual move made by the player with the best move suggested by the chess engine. The chess engine’s suggested moves, while not universally optimal for every position in the dataset, are on average significantly more likely to be the objectively best choice than moves proposed even by the strongest human player. This is due to the substantial disparity in playing strength between modern chess engines and the world’s top human players.

In our main specifications, decision quality is measured by a binary indicator of whether a player makes the best possible move suggested by the chess engine in a given configuration. In robustness checks we also computed the deviation of the evaluation of a player’s move (in terms of pawn units) from the best move identified by the chess engine.

In addition, we use the Maia engine as an alternative benchmark for move prediction. This engine is based on a neural network that is designed to predict human moves rather than to find the best move in a position by brute force computing as embodied in the Stockfish engine (1). The Maia engine provides move recommendations using pattern recognition without traditional sequential tree search. According to the engine website (https://www.maiachess.com/), the Maia engine is trained to reflect a human style of playing chess based on skill, but also exhibits common human biases and mistakes. As an alternative outcome variable, we consider whether the player selects the move predicted by Maia. This benchmark may better reflect the choices players actually consider and could capture strategic dimensions of play that are not represented in a purely computational measure. We use a version of Maia that is trained to predict moves on the level of a player with an ELO rating of 1900.

#### Other variables.

We use the available information to construct three proxy measures for factors that are central to the empirical analysis of the testable hypotheses. First, we use the time budget in terms of time remaining. This is a measure of time pressure and a proxy for the (inverse) time cost for cogitation.

Second, we use a chess engine to compute, for each observed configuration, a measure of the complexity of the configuration. This measure is a proxy for the difficulty of the decision. We construct different measures of complexity. The baseline measure uses the fact that the more complex the configuration, the more nodes the chess engine needs to search in the game-tree arising from a given position to achieve a particular predetermined search depth of moves. The number of nodes needed for the engine to compute the best strategy for the next *n* moves ahead therefore serves as a measure of the complexity of computing the best continuation in a given configuration. As a baseline measure of complexity, we use the number of nodes computed by the chess engine to reach a search-depth of 22 moves. In particular, we use the number of mega-nodes (number of nodes divided by 1,000,000) as our baseline measure of complexity. Modern engines like STOCKFISH calculate approximately 10 to 100 million nodes per second on standard personal computing hardware. The construction of alternative complexity measures leverages the engine’s incremental analysis at different search depths. As first alternative measure (“depth best move”), we compute a measure that identifies at which search depth the engine’s final best move recommendation first appears. Positions in which the best move is identified early (i.e., at a low depth) are considered less complex. Specifically,

[1] $$\begin{array}{c} \text{Depth of Best}{\text{Move}}_{i}=\;\min\left\{d\in \left\{1,2,...,22\right\}:\;m_{i,d}=\;m_{i,22}\right\}, \end{array}$$

where $m_{i,d}$ is the best move recommended by the engine at depth *d* for position *i*, and $m_{i,22}$ is the final best move at maximum depth 22. This measure reflects the fact that in some configurations the best move does not require deep calculations (e.g., when a player is in check). A second alternative measure of complexity is based on the stability or variability of positional evaluations as the engine searches to increasing depths. According to this measure (“absolute change in evaluation over depths”), positions with stable evaluations across different depths are considered less complex than those with volatile evaluations. Specifically, the measure computes the mean absolute change in position evaluation as the engine searches from depth 1 to 22:

[2] $$\begin{array}{c} \text{Abs. Change in}\;{\text{Evaluation}}_{i}=\frac{1}{21}\sum_{d=1}^{21}\left|e_{i,d}-e_{i,22}\right|, \end{array}$$

where $e_{i,d}$ is the position evaluation at depth *d* for position *i*. In addition, we construct measures of complexity based on information obtained from the Maia engine. Particularly, we consider whether the player plays the best move suggested by the Maia engine, whether the best move according to the Stockfish engine coincides with the prediction of the Maia engine, the quality of the move suggested by the Maia engine relative to the best move suggested by the Stockfish engine (computed as the difference in a log-modulus transformation).

In addition to providing alternative measures for complexity, these measures help assess complexity of positions according to traditional search-baed approaches and according to pattern recognition approaches that mimic human play, which might have different implications for the association between decision time and decision quality.

Third, we compute the evaluation gap, computed as the difference between the evaluation of the best continuation as stipulated by the chess engine and the second-best continuation. The larger this difference, the smaller the difficulty of discerning the best move from the other move alternatives (specifically, the second-best alternative) available in a given configuration. *SI Appendix*, Table A2 documents the descriptive statistics of the move-by-move data used in the analysis.

## Supplementary Material

Appendix 01 (PDF)

## Data Availability

A replication package with csv data and a script generating all tables and figures has been deposited in Harvard DataVerse (54).

## Footnotes

^*^Corresponding estimates for time budget, computational complexity, and evaluation gap likewise do not reveal substantial nonlinearities in their associations with decision quality (*SI Appendix*, Fig. A2).

^†^In the context of chess, recent work uses a similar measure, the depth to mate in perfectly solvable endgame positions, as a proxy for the depth and width of the decision tree arising from a position (34).

^‡^Most existing treatments of the drift-diffusion model consider choices between two alternatives (see, e.g., refs. 43 and 49), but the characterization extends to all choice pairs in the set of feasible choices (see ref, 48, for details). Recent work has generalized the model to multiple alternatives (see, e.g., ref. 50) without affecting the main implications relevant for this study.

^§^Recent work has shown that bounded accumulation models have an analogous representation as a random utility model of discrete choice (30). An important implication of this equivalence is that the observed distribution of decisions depends critically on the decision times.

^¶^According to the regulations by the International Chess Federation FIDE, each player must have at least 120 min for a game to be rated for determining the world ranking, assuming the game lasts 60 moves per player. The standard time control regime suggested by the International Chess Federation FIDE is 90 min per player per game plus 30 seconds added to each player’s time budget for each move played; additional 30 min are added to each player’s time budget after each player has played 40 moves (see https://handbook.fide.com, last accessed August 1, 2025).

^#^Source: https://handbook.fide.com/chapter/B022024 and https://handbook.fide.com/chapter/B02RBRegulations2024 (last accessed August 1, 2025).

^‖^After a take-over, www.chess24.com has been deactivated in January 2024 by its new owner www.chess.com.

^**^The ELO rating represents a method for calculating the relative playing strength of players (invented by the Hungarian mathematician Arpad Elo). According to international conventions, an ELO rating of at least 2,500 is a requirement for being awarded the title of International Grandmaster (this requirement has to be fulfilled once during a career, but does not have to be maintained to keep the title, see https://handbook.fide.com/chapter/B01Regulations2017, last accessed January 31, 2024).

^††^We also excluded games with decision times that are likely to be mismeasured (such as negative decision times or times of several hours devoted to a single move) and all games from tournaments that included multiple games with apparently inconsistent decision time data.

^‡‡^We use the version STOCKFISH 17 whose estimated strength of play is equivalent to an ELO of 3,644 points according to https://computerchess.org.uk/ccrl/4040/index.html (accessed on September 15, 2025).

^§§^Information from https://ratings.fide.com (accessed on October 29, 2025).

^¶¶^Most modern chess engines are based on domain-specific algorithmic heuristics that were developed specifically to search the sequential game-tree arising from a given configuration. In particular, these chess engines use an enhanced version of the min-max algorithm that disregards branches of the search tree that have already been found to be dominated. This reduces the search-space without impacting the engine’s final choice of the best move (https://www.chessprogramming.org/Alpha-Beta, last visited October 29, 2025). Recently, more general machine learning techniques in the form of neural networks have been embodied in chess engines such as Google’s nonpublic engine AlphaZero (53), the open-source engine LeelaChessZero, or the Maia Chess project, which trains a neural network designed to capture a human-like style of playing. We use a version of Stockfish 17 that searches the game tree using the standard approach of Alpha-Beta search and evaluates the end positions at the end nodes using a neural network trained on a large database of preevaluated chess positions.

^##^This measure is relative. Positive numbers indicate an advantage for the white pieces, negative numbers indicate an advantage for the black pieces. For example, if the evaluation is −1.00 pawn units, black is better “as if one pawn up.”
