# Supplementary material for: Speed and quality of complex strategic decisions
Source: Proc Natl Acad Sci U S A. 2026 May 13;123(20):e2531472123. doi: 10.1073/pnas.2531472123 (PMC13187726; doi:10.1073/pnas.2531472123)
Supplement: Supplementary file 1 — Appendix 01 (PDF) [file pnas.2531472123.sapp.pdf]

Appendix: Supporting Information

Tables

Table A1. Tournaments included in Dataset

(a) Classical Games

Tournament	Games	Moves
10th Tal Memorial 2016	38	2,380
20th Vidmar Memorial 2016	44	2,721
85th Ukrainian Championship 2016	30	1,355
Armenian Championship 2016-2017	41	2,796
Belarusian Championship 2017	10	395
Dortmund Sparkassen Chess Meeting 2015-2016	53	3,264
Dutch Championship 2015	11	492
FIDE Grand Prix 2014-2015	254	11,877
French Championship 2016	17	517
GRENKE Chess Classic 2014	13	542
LOTTO Polish Championship 2016	36	2,501
Lake Sevan 2016	38	2,464
London Chess Classic 2016	43	1,997
Monte Carlo FIDE Women's Grand Prix 2015	22	1,409
Norway Chess 2014-2016	114	6,221
Poikovsky 2015-2016	84	3,988
Russian Championships 2014-2017	94	4,961
Shamkir Chess 2014-2015	78	3,950
Sinquefield Cup 2016	40	1,968
Tata Steel 2014-2017	338	19,318
U.S. Championship 2015-2016	99	5,517
Total	1,497	80,633

(b) Rapid Games

Tournament	Games	Moves
FIDE World Rapid Championship 2015-2016	1,007	62,825
Total	1,007	62,825

(c) Blitz Games

Tournament	Games	Moves
FIDE World Blitz Championship 2015-2016	1,181	73,945
Total	1,181	73,945

DRAFT

Note: This table shows an overview of tournaments included in our dataset. Classical tournaments include elite single round-robin tournaments played in 2014-2017 with a time budget of at least 120 minutes per player. For Rapid and blitz tournaments, we selected the FIDE Blitz and Rapid World Championships 2015 - 2016 as they provide a substantial prize fund comparable to classical tournaments. Games with erroneous decision times (such as negative decision times or times of several hours for a single move) were excluded. Tournaments with fewer than 10 games and tournaments with inconsistent time controls within the same event were also excluded. The data are restricted to games where both players have at least an ELO rating of 2,500 and in which moves beyond move 15 are played. The data is based on broadcasts of the tournaments on chess24.com.

Table A2. Descriptive Statistics - Move Level

(a) Classical Games

Statistic	N	Mean	St. Dev.	Min	Max
Decision Quality					
Best move (dummy)	80,633	0.59	0.49	0	1
Move quality (log-modulus)	80,633	−0.08	0.34	−5.79	5.79
Decision Time					
Time spent on move (min.)	80,633	2.79	4.30	0.00	63.02
Time Budget					
Remaining time (min.)	80,633	34.39	26.98	0.02	141.65
Time pressure phase before move 40	80,633	0.25	0.43	0	1
Complexity					
N Mega-nodes computed (log)	80,633	15.23	0.68	6.77	20.88
N moves possible	80,633	30.01	11.80	2	67
Depth of best move	80,633	5.39	5.61	1	22
Abs. Change in Evaluation over Depths	80,633	0.78	9.64	0.00	324.82
MAIA move is best move (dummy)	80,633	0.39	0.49	0	1
MAIA move quality (log-modulus)	80,633	−0.27	0.54	−5.81	0.96
Evaluation Gap					
Distance second best move (log)	80,633	0.36	0.64	0.00	5.83
Standard deviation evaluation 6 best moves	80,633	4.33	24.46	0.00	326.94
Controls					
Elo Player	80,633	2,699.09	81.89	2,500	2,881

(b) Rapid Games

Statistic	N	Mean	St. Dev.	Min	Max
Decision Quality					
Best move (dummy)	62,825	0.54	0.50	0	1
Move quality (log-modulus)	62,825	−0.13	0.47	−5.79	5.78
Decision Time					
Time spent on move (min.)	62,825	0.45	0.68	0.00	9.87
Time Budget					
Remaining time (min.)	62,825	4.72	4.28	0.10	18.02
Time pressure phase before move 40	62,825	0.00	0.00	0	0
Complexity					
N Mega-nodes computed (log)	62,825	15.22	0.70	5.11	20.76
N moves possible	62,825	29.18	12.11	2	63
Depth of best move	62,825	5.38	5.62	1	22
Abs. Change in Evaluation over Depths	62,825	1.29	14.25	0.00	323.59
MAIA move is best move (dummy)	62,825	0.39	0.49	0	1
MAIA move quality (log-modulus)	62,825	−0.30	0.61	−5.82	2.10
Evaluation Gap					
Distance second best move (log)	62,825	0.38	0.67	0.00	5.83
Standard deviation evaluation 6 best moves	62,825	5.61	28.17	0.00	245.35
Controls					
Elo Player	62,825	2,662.76	85.57	2,501	2,906

DRAFT

(c) Blitz Games

Statistic	N	Mean	St. Dev.	Min	Max
Decision Quality					
Best move (dummy)	73,945	0.48	0.50	0	1
Move quality (log-modulus)	73,945	−0.20	0.61	−6.48	5.79
Decision Time					
Time spent on move (min.)	73,945	0.09	0.12	0.00	2.98
Time Budget					
Remaining time (min.)	73,945	0.81	0.73	0.00	3.35
Time pressure phase before move 40	73,945	0.00	0.00	0	0
Complexity					
N Mega-nodes computed (log)	73,945	15.25	0.74	4.88	19.72
N moves possible	73,945	29.28	12.09	2	64
Depth of best move	73,945	5.34	5.68	1	22
Abs. Change in Evaluation over Depths	73,945	2.04	18.84	0.00	324.82
MAIA move is best move (dummy)	73,945	0.40	0.49	0	1
MAIA move quality (log-modulus)	73,945	−0.31	0.64	−6.48	5.79
Evaluation Gap					
Distance second best move (log)	73,945	0.42	0.72	0.00	6.48
Standard deviation evaluation 6 best moves	73,945	5.92	28.82	0.00	462.34
Controls					
Elo Player	73,945	2,652.02	88.76	2,500	2,914

Note: Descriptive statistics for subsamples including only classical, rapid and blitz games. The variable $Move\ Quality$ is calculated as $sign(d) \cdot \log(|d|+1)$, where d measures the change in the evaluation by the Stockfish engine before and after a move has been played in a position in pawn units. The variable N moves possible represents the number of possible legal moves in a given configuration. The variable Distance second best move is computed as $ln(d+1)$ where d is the absolute difference between the evaluation of the best and the second best move in terms of pawn units as given by the chess engine. The variable Standard deviation evaluation 6 best moves is calculated as the standard deviation of the evaluation of the chess positions according to the chess engine after each of the six best moves in a chess position would have been played in a given position. Positions with only one viable move option have been excluded from dataset.

Table A3. Alternative Measures for Complexity - Standardized

	Dependent Variable: Best Move (Dummy)								
	Subset: Classical Games								
	(1)	(2)	(3)	(4)	(5)	(6)	(7)	(8)	(9)
Decision Time									
Time spent on move (min.)	-0.018***	-0.018***	-0.015***	-0.018***	-0.015***	-0.015***	-0.018***	-0.012***	-0.014***
	(0.0005)	(0.0005)	(0.0005)	(0.0005)	(0.0005)	(0.0005)	(0.0005)	(0.0005)	(0.0005)
Time Budget									
Remaining time (min.)	0.0008***	0.0007***	0.0007***	0.0008***	0.0005***	0.0005***	0.0008***	0.0006***	0.0007***
	(0.0002)	(0.0002)	(0.0002)	(0.0002)	(0.0002)	(0.0002)	(0.0002)	(0.0002)	(0.0002)
Complexity (Standardized)									
N Mega-nodes (log, std.)	-0.020***							-0.003	-0.006**
	(0.003)							(0.003)	(0.003)
N Moves possible (std.)		-0.025***						-0.007***	-0.008***
		(0.003)						(0.003)	(0.003)
Depth Best Move (std.)			-0.141***					-0.111***	-0.140***
			(0.002)					(0.002)	(0.002)
Abs. Change in Evaluation (std.)				-0.010***				-0.007***	-0.007***
				(0.003)				(0.002)	(0.002)
Maia Move Played (dummy)					0.174***			0.079***	
					(0.004)			(0.005)	
Best Move = Maia Move (dummy)						0.245***		0.178***	
						(0.004)		(0.006)	
Maia Move Quality (log-modulus, std.)							0.014***	-0.057***	-0.0008
							(0.002)	(0.003)	(0.002)
Evaluation Gap									
Distance second best move (log)	0.197***	0.199***	0.156***	0.206***	0.174***	0.156***	0.208***	0.096***	0.154***
	(0.004)	(0.004)	(0.004)	(0.004)	(0.004)	(0.004)	(0.004)	(0.003)	(0.004)
Move Observations	80633	80633	80633	80633	80633	80633	80633	80633	80633
Game Observations	1497	1497	1497	1497	1497	1497	1497	1497	1497
Player-Game Fixed Effects	Yes	Yes	Yes	Yes	Yes	Yes	Yes	Yes	Yes
Control Move Number	Yes	Yes	Yes	Yes	Yes	Yes	Yes	Yes	Yes
Control Evaluation Position	Yes	Yes	Yes	Yes	Yes	Yes	Yes	Yes	Yes

DRAFT

Note: Only classical games included in this analysis. The table presents OLS estimates. The variable `Depth Best Move` is computed as the search depth of Stockfish at which the final best move is for the first time given as the best move. The variable `N Moves possible` is computed as the number of moves available to the player in the current position. The variable `Abs. Change Evaluation` is computed as the absolute value of the difference between the evaluation of the current position at search depth 1 and the final search depth of 22. The variable `Maia Move Played` is a dummy variable indicating whether the move played is also the move suggested by the Maia chess engine. The variable `Best Move = Maia Move` indicates whether the best move by Stockfish is the same as the best move suggested by the Maia engine. The variable `Maia Move Quality` is computed as the difference in evaluation of the current move between the best move being played as suggested by Stockfish and the evaluation of the position after the move suggested by Maia is played. The evaluation of the current position is controlled for using two dummy variables indicating favorable (> 0.5 pawn units) or unfavorable (< -0.5 pawn units) positions for the player about to move. Standard errors are clustered at the game level, and significance levels are indicated as follows: *: $p < 0.1$, **: $p < 0.05$, ***: $p < 0.01$.

Table A4. Correlates of Decision Time

	Dependent Variable: Time Spent on Move (minutes)					
	Subset: Classical				Rapid	Blitz
	(1)	(2)	(3)	(4)	(5)	(6)
Time Budget						
Remaining time (min.)	0.093***			0.089***	0.098***	0.108***
	(0.003)			(0.003)	(0.003)	(0.003)
Complexity						
N Mega-nodes computed		0.941***		0.648***	0.074***	0.008***
		(0.034)		(0.033)	(0.005)	(0.0006)
Evaluation Gap						
Distance second best move (log)			-1.123***	-0.865***	-0.101***	-0.012***
			(0.031)	(0.030)	(0.004)	(0.0006)
Move Observations	80633	80633	80633	80633	62825	73945
Game Observations	1497	1497	1497	1497	1007	1181
Player-Game Fixed Effects	Yes	Yes	Yes	Yes	Yes	Yes
Control Move Number	Yes	Yes	Yes	Yes	Yes	Yes
Control Evaluation Position	Yes	Yes	Yes	Yes	Yes	Yes

Note: The table presents OLS estimates. Columns (1) - (4) only include games played with a classical time control; column (5) only games with a rapid time control; and column (6) games with a blitz time control. The evaluation of the current position is controlled for using two dummy variables indicating favorable (> 0.5 pawn units) or unfavorable (< -0.5 pawn units) positions for the player about to move. Standard errors are clustered at the game level, and significance levels are indicated as follows: *: $p < 0.1$, **: $p < 0.05$, ***: $p < 0.01$.

DRAFT

Table A5. Correlates of Decision Time - Alternative Measures

	Dependent Variable: Time Spent on Move (minutes)							
	Subset: Classical Games							
	(1)	(2)	(3)	(4)	(5)	(6)	(7)	(8)
Time Budget								
Remaining time (min.)	0.089***	0.091***	0.088***	0.089***	0.089***	0.089***	0.109***	0.109***
	(0.003)	(0.003)	(0.003)	(0.003)	(0.003)	(0.003)	(0.004)	(0.004)
Complexity (Standardized)								
N Mega-nodes computed (log, std.)	0.442***							0.341***
	(0.022)							(0.037)
N Moves possible (std.)		0.533***						0.437***
		(0.023)						(0.039)
Depth Best Move (std.)			0.431***					0.263***
			(0.016)					(0.020)
Abs. Change in Evaluation (std.)				0.025**				0.024
				(0.012)				(0.016)
Maia Move Played (dummy)					-1.179***			-0.338***
					(0.030)			(0.055)
Best Move = Maia Move (dummy)						-0.952***		-0.079
						(0.031)		(0.075)
Maia Move Quality (log-modulus)							-0.042***	0.026
							(0.016)	(0.017)
Evaluation Gap								
Distance second best move (log)	-0.865***	-0.917***	-0.874***	-1.039***	-0.807***	-0.833***	-0.871***	-0.532***
	(0.030)	(0.030)	(0.027)	(0.030)	(0.026)	(0.027)	(0.059)	(0.056)
Move Observations	80633	80633	80633	80633	80633	80633	46761	46761
Game Observations	1497	1497	1497	1497	1497	1497	1497	1497
Player-Game Fixed Effects	Yes	Yes	Yes	Yes	Yes	Yes	Yes	Yes
Control Move Number	Yes	Yes	Yes	Yes	Yes	Yes	Yes	Yes
Control Evaluation Position	Yes	Yes	Yes	Yes	Yes	Yes	Yes	Yes

DRAFT

Note: Only classical games included in this analysis. The table presents OLS estimates. The variable `N Moves possible` is computed as the number of moves available to the player in the current position. The variable `Depth Best Move` is computed as the search depth of Stockfish at which the final best move is for the first time given as the best move. The variable `Abs. Change Evaluation` is computed as the absolute value of the difference between the evaluation of the current position at search depth 1 and the final search depth of 22. The variable `Maia Move Played` is a dummy variable indicating whether the move played is also the move suggested by the Maia chess engine. The variable `Best Move = Maia Move` indicates whether the best move by Stockfish is the same as the best move suggested by the Maia engine. The variable `Maia Move Quality` is computed as the difference in evaluation of the current move between the best move being played as suggested by Stockfish and the evaluation of the position after the move suggested by Maia is played. All continuous measures have been standardized to allow for comparability. The evaluation of the current position is controlled for using two dummy variables indicating favorable (> 0.5 pawn units) or unfavorable (< -0.5 pawn units) positions for the player about to move. Standard errors are clustered at the game level, and significance levels are indicated as follows: *: $p < 0.1$, **: $p < 0.05$, ***: $p < 0.01$.

Table A6. Results with whether Maia Move played as Outcome variable - Per Subsample

	Dependent Variable: Maia Move Played (Dummy)								
	Subset:								
	Classical			Rapid			Blitz		
	(1)	(2)	(3)	(4)	(5)	(6)	(7)	(8)	(9)
Decision Time									
Time spent on move (min.)	-0.021***	-0.017***	-0.022***	-0.140***	-0.124***	-0.177***	-0.815***	-0.760***	-1.081***
	(0.0005)	(0.0005)	(0.0006)	(0.003)	(0.003)	(0.005)	(0.023)	(0.023)	(0.027)
Time Budget									
Remaining time (min.)		0.001***	0.001***		0.011***	0.009***		0.057***	0.039***
		(0.0002)	(0.0002)		(0.001)	(0.001)		(0.008)	(0.008)
Complexity									
N Mega-nodes computed		-0.057***	-0.056***		-0.050***	-0.044***		-0.053***	-0.052***
		(0.004)	(0.004)		(0.005)	(0.005)		(0.004)	(0.004)
Evaluation Gap									
Distance second best move (log)		0.160***	0.159***		0.128***	0.121***		0.100***	0.095***
		(0.005)	(0.005)		(0.005)	(0.006)		(0.004)	(0.004)
Interactions									
Time spent x Remaining time			0.0002***			0.012***			0.403***
			(0.00002)			(0.0008)			(0.025)
Time spent x N Mega-nodes			0.0001			0.021***			0.024
			(0.0009)			(0.007)			(0.039)
Time spent x Dist. second best			0.001			-0.006			-0.054
			(0.002)			(0.010)			(0.038)
Move Observations	80633	80633	80633	62825	62825	62825	73945	73945	73945
Game Observations	1497	1497	1497	1007	1007	1007	1181	1181	1181
Player-Game Fixed Effects	Yes	Yes	Yes	Yes	Yes	Yes	Yes	Yes	Yes
Control Move Number	Yes	Yes	Yes	Yes	Yes	Yes	Yes	Yes	Yes
Control Evaluation Position	Yes	Yes	Yes	Yes	Yes	Yes	Yes	Yes	Yes

Note: The table presents OLS estimates. The dependent variable `Maia Move Played` is dummy variable indicating whether the move predicted by the Maia engine was played. The engine is trained to predict the move played by a player with Elo 1900. Columns (1) - (3) only include games played with a classical time control; columns (4) - (6) only games with a rapid time control; and columns (7) - (9) games with a blitz time control. The evaluation of the current position is controlled for using two dummy variables indicating favorable (> 0.5 pawn units) or unfavorable (< -0.5 pawn units) positions for the player about to move. All independent variables have been demeaned, allowing main effects to be interpreted as marginal effects at the mean when interaction terms are present. Standard errors are clustered at the game level, and significance levels are indicated as follows: *: $p < 0.1$, **: $p < 0.05$, ***: $p < 0.01$.

DRAFT

Table A7. Baseline Results with Interactions - Per Subsample by whether Best Move equal Best Maia Move

	Dependent Variable: Best Move (Dummy)					
	Subset: Classical					
	Best Move = Maia Move			Best Move = NOT Maia Move		
	(1)	(2)	(3)	(4)	(5)	(6)
Decision Time						
Time spent on move (min.)	-0.029***	-0.025***	-0.032***	-0.013***	-0.011***	-0.011***
	(0.001)	(0.001)	(0.001)	(0.0006)	(0.0006)	(0.0009)
Time Budget						
Remaining time (min.)		0.001***	0.001***		0.0002	0.00008
		(0.0002)	(0.0002)		(0.0003)	(0.0003)
Complexity						
N Mega-nodes computed (log)		-0.011**	-0.017***		-0.026***	-0.030***
		(0.005)	(0.006)		(0.005)	(0.005)
Evaluation Gap						
Distance second best move (log)		0.104***	0.150***		0.262***	0.283***
		(0.003)	(0.009)		(0.011)	(0.011)
Interactions						
Time spent x Remaining time			0.0004***			0.0002***
			(0.00003)			(0.00002)
Time spent x N Mega-nodes			-0.004***			-0.004***
			(0.002)			(0.001)
Time spent x Dist. second best			0.021***			0.014***
			(0.003)			(0.002)
Move Observations	31575	31575	31575	49058	49058	49058
Game Observations	1493	1493	1493	1495	1495	1495
Player-Game Fixed Effects	Yes	Yes	Yes	Yes	Yes	Yes
Control Move Number	Yes	Yes	Yes	Yes	Yes	Yes
Control Evaluation Position	Yes	Yes	Yes	Yes	Yes	Yes

DRAFT

Note: The table presents OLS estimates. Only games using a classical time control are included. Columns (1) - (3) give results on the subset of positions in which the best move as given by the Stockfish engine coincides with the best move predicted my the Maia engine. Columns (4) - (6) give results for positions in which this is not the case. All independent variable are demeaned. The evaluation of the current position is controlled for using two dummy variables indicating favorable (> 0.5 pawn units) or unfavorable (< -0.5 pawn units) positions for the player about to move. All independent variables have been demeaned, allowing main effects to be interpreted as marginal effects at the mean when interaction terms are present. Standard errors are clustered at the game level, and significance levels are indicated as follows: *: $p < 0.1$, **: $p < 0.05$, ***: $p < 0.01$.

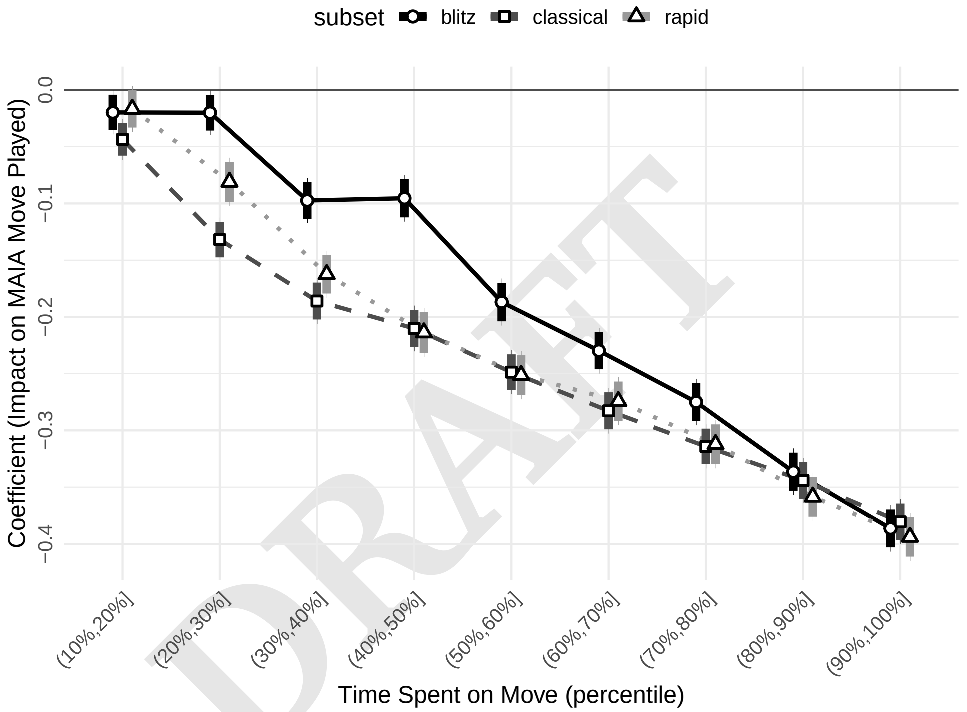

Fig. A1. Flexibly Estimated Coefficients of Impact Time Spent on Best Move by Maia played by Frequency Deciles

Note: Plot shows flexibly estimated coefficients for the effect of time spent on move quality as approximated by Maia engine across classical, rapid, and blitz time controls. Time spent on move is divided into decile bins based on the distribution within each time control format. The [0%, 10%] percentile is the omitted baseline category. Coefficients represent the change in probability of playing the best move as predicted by the Maia engine for each time decile relative to the baseline. Each regression also includes the variables measuring complexity, available time budget and evaluation gap as in Table 1 as well as player-game fixed effects, move number fixed effects, and controls for position evaluation (better/worse position indicators). Error bars show 95% confidence intervals based on standard errors clustered at the game level.

DRAFT

Table A8. Determinants of Decision Quality - Robustness - Intensive Margin

	Dependent Variable: Move Quality (log-modulus)								
	Subset:								
	Classical			Rapid			Blitz		
	(1)	(2)	(3)	(4)	(5)	(6)	(7)	(8)	(9)
Decision Time									
Time spent on move (min.)	-0.007***	-0.006***	-0.011***	-0.050***	-0.057***	-0.092***	-0.361***	-0.426***	-0.690***
	(0.0003)	(0.0003)	(0.0007)	(0.003)	(0.003)	(0.006)	(0.017)	(0.020)	(0.038)
Time Budget									
Remaining time (min.)		0.0009***	0.0008***		0.012***	0.011***		0.065***	0.050***
		(0.0001)	(0.0001)		(0.002)	(0.002)		(0.012)	(0.012)
Complexity									
N Mega-nodes computed		-0.040***	-0.041***		-0.062***	-0.061***		-0.069***	-0.071***
		(0.004)	(0.004)		(0.007)	(0.007)		(0.007)	(0.007)
Evaluation Gap									
Distance second best move (log)		0.011	-0.022**		-0.043***	-0.079***		-0.089***	-0.124***
		(0.006)	(0.009)		(0.010)	(0.015)		(0.012)	(0.016)
Interactions									
Time spent x Remaining time			0.0001***			0.005***			0.247***
			(0.00001)			(0.0007)			(0.025)
Time spent x N Mega-nodes			-0.003***			-0.005			-0.061
			(0.0005)			(0.008)			(0.050)
Time spent x Dist. second best			-0.015***			-0.111***			-0.714***
			(0.003)			(0.028)			(0.207)
Move Observations	80633	80633	80633	62825	62825	62825	73945	73945	73945
Game Observations	1497	1497	1497	1007	1007	1007	1181	1181	1181
Player-Game Fixed Effects	Yes	Yes	Yes	Yes	Yes	Yes	Yes	Yes	Yes
Control Move Number	Yes	Yes	Yes	Yes	Yes	Yes	Yes	Yes	Yes
Control Evaluation Position	Yes	Yes	Yes	Yes	Yes	Yes	Yes	Yes	Yes

DRAFT

Note: OLS estimates. The dependent variable Move Quality (Log-Modulus) is a measure of the distance of the actual move played from the optimal move stipulated by the chess engine, in terms of the log modulus transformation of pawn units, such that $Move\ Quality = sign(d) \cdot \ln(|d| + 1)$, where d measures the change in the evaluation by the Stockfish engine before and after a move has been played in a position in pawn units. The evaluation of the current position is controlled for using two dummy variables indicating whether the current position is evaluated as better (> 0.5 pawn units) or worse (< -0.5 pawn units) for the player to move. All independent variables have been demeaned, allowing main effects to be interpreted as marginal effects at the mean when interaction terms are present. Standard errors are clustered on the game level. *: $p < 0.1$, **: $p < 0.05$, ***: $p < 0.01$.

Table A9. Determinants of Decision Quality - Robustness - Best Move Weak Engine

	Dependent Variable: Best Move Weak Engine (Dummy)								
	Subset:								
	Classical			Rapid			Blitz		
	(1)	(2)	(3)	(4)	(5)	(6)	(7)	(8)	(9)
Decision Time									
Time spent on move (min.)	-0.023***	-0.017***	-0.020***	-0.135***	-0.106***	-0.145***	-0.657***	-0.544***	-0.735***
	(0.0005)	(0.0005)	(0.0007)	(0.003)	(0.003)	(0.005)	(0.019)	(0.018)	(0.025)
Time Budget									
Remaining time (min.)		0.0004*	0.0002		0.003*	0.0008		0.021**	0.010
		(0.0002)	(0.0002)		(0.002)	(0.002)		(0.008)	(0.008)
Complexity									
N Mega-nodes computed (log)		-0.033***	-0.038***		-0.047***	-0.047***		-0.058***	-0.061***
		(0.004)	(0.004)		(0.005)	(0.005)		(0.004)	(0.004)
Evaluation Gap									
Distance second best move (log)		0.203***	0.228***		0.183***	0.188***		0.153***	0.149***
		(0.005)	(0.007)		(0.005)	(0.007)		(0.005)	(0.005)
Interactions									
Time spent x Remaining time			0.0003***			0.012***			0.278***
			(0.00002)			(0.0008)			(0.022)
Time spent x N Mega-nodes			-0.004***			-0.005			-0.093***
			(0.0009)			(0.007)			(0.035)
Time spent x Dist. second best			0.013***			0.028**			-0.041
			(0.002)			(0.013)			(0.047)
Move Observations	80633	80633	80633	62825	62825	62825	73945	73945	73945
Game Observations	1497	1497	1497	1007	1007	1007	1181	1181	1181
Player-Game Fixed Effects	Yes	Yes	Yes	Yes	Yes	Yes	Yes	Yes	Yes
Control Move Number	Yes	Yes	Yes	Yes	Yes	Yes	Yes	Yes	Yes
Control Evaluation Position	Yes	Yes	Yes	Yes	Yes	Yes	Yes	Yes	Yes

DRAFT

Note: OLS estimates. The dependent variable is a dummy variable indicating whether the best move was played as suggested by a weak chess engine that is constructed to be of comparable strength as the average player in the dataset. For this purpose, for classical games Stockfish was restricted to use a search depth of 9, for rapid games a search depth of 8, and for blitz a search depth of 7. These search depths were picked as at these settings the restricted Stockfish engine has similar agreements in terms of the best move suggested with the full strength Stockfish as the players in the respective subsets of the data have. The evaluation of the current position is controlled for using two dummy variables indicating whether the current position is evaluated as better (> 0.5 pawn units) or worse (< -0.5 pawn units) for the player to move. All independent variables have been demeaned, allowing main effects to be interpreted as marginal effects at the mean when interaction terms are present. Standard errors are clustered on the game level. *: $p < 0.1$, **: $p < 0.05$, ***: $p < 0.01$.

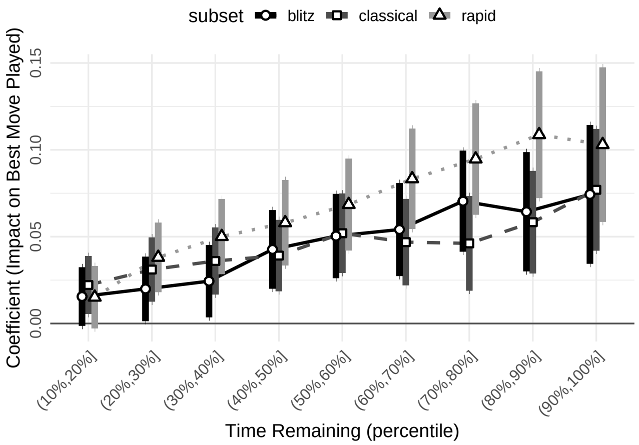

(a) Time Budget

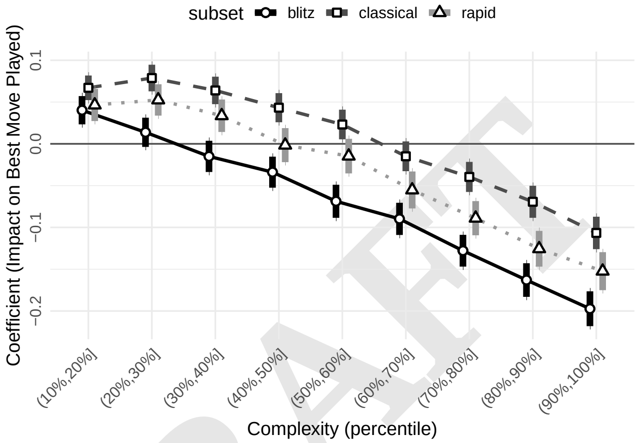

(b) Complexity

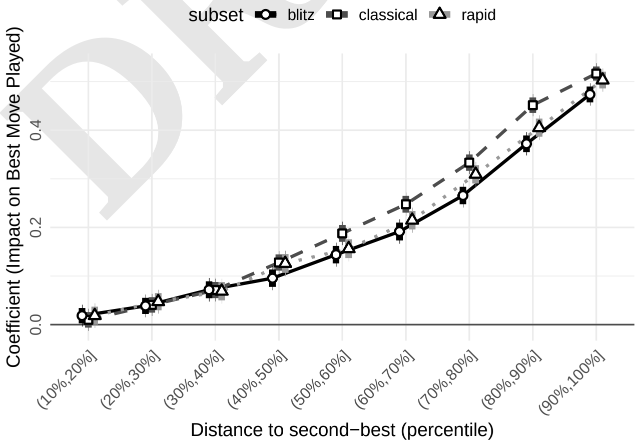

(c) Evaluation Gap

DRAFT

Fig. A2. Other Determinants of Decision Quality - Flexible Specifications

Note: Plot shows flexibly estimated coefficients for the effect of the determinants of decision quality except time spent on move across classical, rapid, and blitz time controls. Variables are divided into decile bins based on the distribution within each time control format. The [0%, 10%] percentile is the omitted baseline category. Coefficients represent the change in probability of playing the best move for each decile relative to the baseline. Each regression also includes the variables measuring complexity, available time budget and evaluation gap as in table **??** as well as player-game fixed effects, move number fixed effects, and controls for position evaluation (better/worse position indicators). Error bars show 95% confidence intervals based on standard errors clustered at the game level.

Figures

3

DRAFT

Table A10. Robustnesss - Accounting for Decision Time and Quality in Previous Moves

	Dependent Variable: Best Move (Dummy)								
	Subset: Classical			Rapid			Blitz		
	(1)	(2)	(3)	(4)	(5)	(6)	(7)	(8)	(9)
Decision Time									
Time spent on move (min.)	-0.018***	-0.019***	-0.019***	-0.108***	-0.112***	-0.114***	-0.550***	-0.568***	-0.575***
	(0.0005)	(0.0005)	(0.0005)	(0.003)	(0.004)	(0.004)	(0.018)	(0.020)	(0.019)
Time Budget									
Remaining time (min.)	0.0008***	0.0007***	0.0006***	0.006***	0.006***	0.006***	0.022***	0.019**	0.022***
	(0.0002)	(0.0002)	(0.0002)	(0.002)	(0.002)	(0.002)	(0.008)	(0.008)	(0.008)
Complexity									
N Mega-nodes computed (log)	-0.029***	-0.026***	-0.027***	-0.052***	-0.049***	-0.049***	-0.067***	-0.064***	-0.064***
	(0.004)	(0.004)	(0.004)	(0.005)	(0.005)	(0.005)	(0.004)	(0.004)	(0.004)
Evaluation Gap									
Distance second best move (log)	0.197***	0.194***	0.193***	0.174***	0.171***	0.170***	0.152***	0.149***	0.148***
	(0.004)	(0.004)	(0.004)	(0.005)	(0.005)	(0.005)	(0.005)	(0.005)	(0.005)
Decision Quality Previous Moves									
Best Move (previous own move)		0.021***	0.022***		0.033***	0.034***		0.025***	0.027***
		(0.004)	(0.004)		(0.004)	(0.004)		(0.004)	(0.004)
Best Move (previous opponent's move)		0.009**	0.017***		0.008*	0.012***		0.016***	0.020***
		(0.003)	(0.004)		(0.004)	(0.004)		(0.004)	(0.004)
Decision Time Previous Moves									
Time spent on move (previous own move)			-0.002***			0.0001			0.032*
			(0.0004)			(0.003)			(0.017)
Time spent on move (previous opponent's move)			0.005***			0.021***			0.116***
			(0.0004)			(0.003)			(0.017)
Move Observations	80633	77641	77641	62825	60812	60812	73945	71583	71583
Game Observations	1497	1497	1497	1007	1007	1007	1181	1181	1181
Player-Game Fixed Effects	Yes	Yes	Yes	Yes	Yes	Yes	Yes	Yes	Yes
Control Move Number	Yes	Yes	Yes	Yes	Yes	Yes	Yes	Yes	Yes
Control Evaluation Position	Yes	Yes	Yes	Yes	Yes	Yes	Yes	Yes	Yes

Note: The table presents OLS estimates. Columns (1), (4) and (7) replicate the main specification without interactions from Table **??** for direct comparability. The evaluation of the current position is controlled for using two binary variables indicating favorable (> 0.5 pawn units) or unfavorable (< -0.5 pawn units) positions for the player about to move. All independent variables have been demeaned, allowing main effects to be interpreted as marginal effects at the mean when interaction terms are present. Standard errors are clustered at the game level, and significance levels are indicated as follows: *: $p < 0.1$, **: $p < 0.05$, ***: $p < 0.01$.

Table A11. Determinants of Decision Time - Robustness - Previous Own Moves and Opponent

	Dependent Variable: Time Spent on Move (min.)								
	Subset:								
	Classical			Rapid			Blitz		
	(1)	(2)	(3)	(4)	(5)	(6)	(7)	(8)	(9)
Time Budget									
Remaining time (min.)	0.089***	0.088***	0.088***	0.098***	0.099***	0.099***	0.108***	0.109***	0.109***
	(0.003)	(0.003)	(0.003)	(0.003)	(0.004)	(0.004)	(0.003)	(0.003)	(0.003)
Complexity									
N Mega-nodes computed (log)	0.648***	0.587***	0.572***	0.074***	0.069***	0.068***	0.008***	0.007***	0.008***
	(0.033)	(0.031)	(0.031)	(0.005)	(0.005)	(0.005)	(0.0006)	(0.0006)	(0.0006)
Evaluation Gap									
Distance second best move (log)	-0.865***	-0.829***	-0.837***	-0.101***	-0.098***	-0.100***	-0.012***	-0.012***	-0.012***
	(0.030)	(0.029)	(0.029)	(0.004)	(0.004)	(0.004)	(0.0006)	(0.0005)	(0.0006)
Decision Quality Previous Moves									
Best Move (previous own move)		0.048*	0.110***		0.018***	0.023***		-0.0001	-0.00002
		(0.029)	(0.029)		(0.005)	(0.005)		(0.0008)	(0.0008)
Best Move (previous opponent's move)		-0.484***	-0.323***		-0.045***	-0.029***		-0.001	0.0006
		(0.031)	(0.031)		(0.005)	(0.005)		(0.0008)	(0.0008)
Decision Time Previous Moves									
Time spent on move (previous own move)			-0.0007			0.002			-0.0002
			(0.005)			(0.006)			(0.005)
Time spent on move (previous opponent's move)			0.100***			0.070***			0.048***
			(0.006)			(0.007)			(0.009)
Move Observations	80633	77641	77641	62825	60812	60812	73945	71583	71583
Game Observations	1497	1497	1497	1007	1007	1007	1181	1181	1181
Player-Game Fixed Effects	Yes	Yes	Yes	Yes	Yes	Yes	Yes	Yes	Yes
Control Move Number	Yes	Yes	Yes	Yes	Yes	Yes	Yes	Yes	Yes
Control Evaluation Position	Yes	Yes	Yes	Yes	Yes	Yes	Yes	Yes	Yes

DRAFT

Note: OLS estimates. Columns (1), (4) and (7) show for comparision the main specification from table A4. The evaluation of the current position is controlled for using two dummy variables indicating whether the current position is evaluated as better (> 0.5 pawn units) or worse (< -0.5 pawn units) for the player to move. All independent variables have been demeaned, allowing main effects to be interpreted as marginal effects at the mean when interaction terms are present. Standard errors are clustered on the game level. *: $p < 0.1$, **: $p < 0.05$, ***: $p < 0.01$.

Table A12. Determinants of Decision Time - Accounting for Surprise Moves

	Dependent Variable: Time Spent on Move (min.)				
	Subset: Classical				
	(1)	(2)	(3)	(4)	(5)
Time Budget					
Remaining time (min.)	0.088***	0.088***	0.088***	0.088***	0.089***
	(0.003)	(0.003)	(0.003)	(0.003)	(0.003)
Complexity					
N Mega-nodes computed (log)	0.563***	0.565***	0.599***	0.593***	0.605***
	(0.031)	(0.031)	(0.032)	(0.032)	(0.032)
Evaluation Gap					
Distance second best move (log)	-0.745***	-0.744***	-0.831***	-0.803***	-0.862***
	(0.027)	(0.028)	(0.029)	(0.028)	(0.030)
Previous Opponent's Move					
Best move played	-0.216***	-0.109***	-0.350***	-1.023***	
	(0.031)	(0.042)	(0.034)	(0.043)	
Maia move played	-1.173***	-0.980***			
	(0.030)	(0.046)			
Search-depth best move			0.040***	-0.010***	
			(0.003)	(0.003)	
Move quality (log)					-0.305***
					(0.058)
Rank move played					0.092***
					(0.009)
Interactions					
Best move X Maia move		-0.298***			
		(0.057)			
Best move X Search-depth				0.127***	
				(0.006)	
Move Observations	79136	79136	79136	79136	79136
Game Observations	1497	1497	1497	1497	1497
Player-Game Fixed Effects	Yes	Yes	Yes	Yes	Yes
Control Move Number	Yes	Yes	Yes	Yes	Yes
Control Evaluation Position	Yes	Yes	Yes	Yes	Yes

DRAFT

Note: OLS estimates. This table explores how 'surprise' moves by an opponent affect a player's decision time. Surprise is measured using several indicators of the opponent's previous move. Moves that are anticipated, such as the Best move played (an indicator for whether the opponent played the engine's top choice) and the Maia move played (an indicator for whether the opponent played the move predicted by the human-like Maia engine), are associated with shorter decision times. Conversely, moves that are harder to anticipate are considered more surprising and lead to longer decision times. These include moves requiring a deeper search to be identified as best (Search-depth best move), lower quality moves (Move quality (log), measured as the change in pawn units), and moves ranked lower among the engine's top choices (Rank move played). The rank of moves not appearing in the list of engine's six best moves is set to 7. The evaluation of the current position is controlled for using two dummy variables indicating whether the current position is evaluated as better (> 0.5 pawn units) or worse (< -0.5 pawn units) for the player to move. Standard errors are clustered at the game level. *: $p < 0.1$, **: $p < 0.05$, ***: $p < 0.01$.

Table A13. Determinants of Best Move - Accounting for Surprise Moves

	Dependent Variable: Best Move (Dummy)				
	Subset: Classical				
	(1)	(2)	(3)	(4)	(5)
Decision Time					
Time spent on move (min.)	-0.017***	-0.017***	-0.018***	-0.018***	-0.018***
	(0.0005)	(0.0005)	(0.0005)	(0.0005)	(0.0005)
Time Budget					
Remaining time (min.)	0.0007***	0.0007***	0.0008***	0.0007***	0.0008***
	(0.0002)	(0.0002)	(0.0002)	(0.0002)	(0.0002)
Complexity					
N Mega-nodes computed (log)	-0.026***	-0.026***	-0.027***	-0.027***	-0.028***
	(0.004)	(0.004)	(0.004)	(0.004)	(0.004)
Evaluation Gap					
Distance second best move (log)	0.193***	0.193***	0.195***	0.195***	0.195***
	(0.004)	(0.004)	(0.004)	(0.004)	(0.004)
Previous Opponent’s Move					
Best move played	0.0009	0.005	0.003	0.027***	
	(0.004)	(0.005)	(0.004)	(0.005)	
Maia move played	0.039***	0.046***			
	(0.004)	(0.006)			
Search-depth best move			-0.002***	-0.00005	
			(0.0003)	(0.0004)	
Move quality (log)					-0.015*
					(0.008)
Rank move played					-0.0008
					(0.001)
Interactions					
Best move X Maia move		-0.011			
		(0.007)			
Best move X Search-depth				-0.004***	
				(0.0007)	
Move Observations	79136	79136	79136	79136	79136
Game Observations	1497	1497	1497	1497	1497
Player-Game Fixed Effects	Yes	Yes	Yes	Yes	Yes
Control Move Number	Yes	Yes	Yes	Yes	Yes
Control Evaluation Position	Yes	Yes	Yes	Yes	Yes

DRAFT

Note: OLS estimates. This table explores how ’surprise’ moves by an opponent affect the quality of a player’s move, measured by whether the player makes the best move. The ’surprise’ variables, which describe the opponent’s previous move, are defined as follows: Best move played is an indicator for whether the opponent played the engine’s top choice. Maia move played indicates if the opponent played the move predicted by the human-like Maia engine. Search-depth best move is the search depth required to identify the opponent’s best move. Move quality (log) is the quality of the opponent’s move measured in pawn units. Rank move played is the rank of the opponent’s move among the engine’s top choices. The rank of moves not appearing in the list of engine’s six best moves is set to 7. The evaluation of the current position is controlled for using two dummy variables indicating whether the current position is evaluated as better (> 0.5 pawn units) or worse (< -0.5 pawn units) for the player to move. Standard errors are clustered on the game level. *: $p < 0.1$, **: $p < 0.05$, ***: $p < 0.01$.

Table A14. Determinants of Decision Quality - No piece or pawn taken in previous position

	Dependent Variable: Best Move (Dummy)					
	Subset:					
	No previous capture or check			No current capture or check		
	Classical	Rapid	Blitz	Classical	Rapid	Blitz
	(1)	(2)	(3)	(4)	(5)	(6)
Decision Time						
Time spent on move (min.)	-0.017***	-0.101***	-0.549***	-0.019***	-0.116***	-0.650***
	(0.0008)	(0.006)	(0.029)	(0.0009)	(0.006)	(0.029)
Time Budget						
Remaining time (min.)	0.0006**	0.005**	0.004	0.0008***	0.006***	0.010
	(0.0002)	(0.002)	(0.010)	(0.0002)	(0.002)	(0.010)
Complexity						
N Mega-nodes computed (log)	-0.027***	-0.045***	-0.061***	-0.033***	-0.049***	-0.063***
	(0.005)	(0.006)	(0.005)	(0.005)	(0.005)	(0.004)
Evaluation Gap						
Distance second best move (log)	0.292***	0.220***	0.148***	0.232***	0.180***	0.131***
	(0.010)	(0.009)	(0.008)	(0.009)	(0.008)	(0.006)
Interactions						
Time spent x Remaining time	0.0003***	0.008***	0.237***	0.0003***	0.007***	0.266***
	(0.00002)	(0.0009)	(0.025)	(0.00002)	(0.0009)	(0.024)
Time spent x N Mega-nodes	-0.003***	-0.018**	-0.086**	-0.002*	0.004	0.009
	(0.001)	(0.009)	(0.042)	(0.001)	(0.008)	(0.039)
Time spent x Dist. second best	0.017***	0.055***	0.166***	0.016***	0.067***	0.198***
	(0.002)	(0.013)	(0.058)	(0.003)	(0.013)	(0.053)
Move Observations	55757	44201	51686	56809	44889	52511
Game Observations	1497	1007	1181	1497	1007	1181
Player-Game Fixed Effects	Yes	Yes	Yes	Yes	Yes	Yes
Control Move Number	Yes	Yes	Yes	Yes	Yes	Yes
Control Evaluation Position	Yes	Yes	Yes	Yes	Yes	Yes

DRAFT

Note:OLS estimates are presented. In columns (1) - (3) only positions in which no piece or pawn has been taken and no check was given in the previous move are included. In columns (4) - (6) only positions in which no piece or pawn has been taken and no check was given in the current position are included. The evaluation of the current position is controlled for using two dummy variables indicating favorable (> 0.5 pawn units) or unfavorable (< -0.5 pawn units) positions for the player to move. All independent variables have been demeaned, allowing main effects to be interpreted as marginal effects at the mean when interaction terms are present. Standard errors are clustered at the game level, and significance levels are indicated as follows: *: $p < 0.1$, **: $p < 0.05$, ***: $p < 0.01$.

Table A15. Results split by Middle Game or Endgame - Per Subsample

	Dependent Variable: Best Move (Dummy)						
	Subset: Classical						
	All Positions	Middlegame Positions			Endgame Positions		
		with Queens	>7 pieces	$<$ move 60	w/o Queens	≤ 7 pieces	$\geq$ move 60
	(1)	(2)	(3)	(4)	(5)	(6)	(7)
Decision Time							
Time spent on move (min.)	-0.021***	-0.020***	-0.020***	-0.020***	-0.021***	-0.037***	-0.040***
	(0.0007)	(0.0009)	(0.0007)	(0.0007)	(0.001)	(0.008)	(0.011)
Time Budget							
Remaining time (min.)	0.0006***	0.001***	0.0007***	0.0006***	0.0003	-0.004	0.004
	(0.0002)	(0.0003)	(0.0002)	(0.0002)	(0.0003)	(0.003)	(0.003)
Complexity							
N Mega-nodes computed (log)	-0.034***	-0.062***	-0.036***	-0.037***	-0.010	-0.009	-0.003
	(0.004)	(0.006)	(0.005)	(0.005)	(0.006)	(0.025)	(0.020)
Evaluation Gap							
Distance second best move (log)	0.239***	0.216***	0.244***	0.244***	0.270***	0.125***	0.148***
	(0.007)	(0.008)	(0.007)	(0.007)	(0.010)	(0.045)	(0.056)
Interactions							
Time spent x Remaining time	0.0003***	0.0003***	0.0003***	0.0003***	0.0003***	0.001***	0.002***
	(0.00002)	(0.00002)	(0.00002)	(0.00002)	(0.00003)	(0.0004)	(0.0003)
Time spent x N Mega-nodes	-0.004***	-0.003**	-0.003***	-0.004***	-0.003**	-0.011	-0.005
	(0.0008)	(0.001)	(0.0008)	(0.0008)	(0.001)	(0.009)	(0.008)
Time spent x Dist. second best	0.021***	0.021***	0.021***	0.020***	0.022***	0.004	0.006
	(0.002)	(0.002)	(0.002)	(0.002)	(0.003)	(0.017)	(0.021)
Move Observations	80633	45301	77316	75012	35332	3317	5621
Game Observations	1497	1343	1497	1497	853	155	196
Player-Game Fixed Effects	Yes	Yes	Yes	Yes	Yes	Yes	Yes
Control Move Number	Yes	Yes	Yes	Yes	Yes	Yes	Yes
Control Evaluation Position	Yes	Yes	Yes	Yes	Yes	Yes	Yes

DRAFT

Note: Models run on subsets of classical time control games split by whether a given chess position can be considered as a middlegame or endgame position. Different definitions are used: a) whether or not any queen is still on the board in columns (2) and (5), b) whether or not more than 7 pieces (overall, including both players, kings and pawns included) are still on the board in columns (3) and (6), c) whether or not a position is played before or after move 60 of a game in columns (4) and (7). The evaluation of the current position is controlled for using two dummy variables indicating favorable (>0.5 pawn units) or unfavorable (<-0.5 pawn units) positions for the player about to move. Standard errors are clustered at the game level, and significance levels are indicated as follows: *: $p<0.1$, **: $p<0.05$, ***: $p<0.01$.

Table A16. Determinants of Decision Quality - Split by overall number of moves in game

	Dependent Variable: Best Move (Dummy)						
	Subset: Classical Games						
	Number of Moves in Game:						
	<21	21-30	31-40	41-50	51-60	61-80	80+
Decision Time							
Time spent on move (min.)	-0.048**	-0.021***	-0.020***	-0.019***	-0.021***	-0.022***	-0.025***
	(0.019)	(0.002)	(0.001)	(0.002)	(0.001)	(0.002)	(0.003)
Time Budget							
Remaining time (min.)	-0.017**	-0.0004	0.0002	0.0007*	0.0007*	0.0009*	0.003***
	(0.007)	(0.0009)	(0.0004)	(0.0004)	(0.0004)	(0.0005)	(0.0007)
Complexity							
N Mega-nodes computed (log)	-0.105**	-0.061***	-0.031***	-0.051***	-0.021**	-0.034***	-0.034*
	(0.048)	(0.017)	(0.009)	(0.008)	(0.009)	(0.010)	(0.017)
Evaluation Gap							
Distance second best move (log)	0.149*	0.202***	0.260***	0.226***	0.248***	0.232***	0.263***
	(0.074)	(0.021)	(0.010)	(0.015)	(0.014)	(0.015)	(0.021)
Interactions							
Time spent x Remaining time	0.0007**	0.0003***	0.0003***	0.0002***	0.0003***	0.0004***	0.0005***
	(0.0003)	(0.00005)	(0.00003)	(0.00004)	(0.00004)	(0.00005)	(0.0001)
Time spent x N Mega-nodes	0.039*	-0.002	-0.003*	-0.003*	-0.003*	-0.005**	-0.006
	(0.019)	(0.003)	(0.002)	(0.001)	(0.002)	(0.002)	(0.004)
Time spent x Dist. second best	0.046	0.016***	0.029***	0.013**	0.021***	0.017***	0.037***
	(0.028)	(0.004)	(0.003)	(0.005)	(0.004)	(0.005)	(0.007)
Move Observations	198	6722	20482	17300	15358	13535	7038
Game Observations	32	285	501	306	198	130	45
Player-Game Fixed Effects	Yes	Yes	Yes	Yes	Yes	Yes	Yes
Control Move Number	Yes	Yes	Yes	Yes	Yes	Yes	Yes
Control Evaluation Position	Yes	Yes	Yes	Yes	Yes	Yes	Yes

DRAFT

Note: Table with OLS estimates splitting data set in game of different length, as measure by the total number of moves by both players. All variables have been demeaned. The evaluation of the current position is controlled for using two dummy variables indicating whether the current position is evaluated as better (> 0.5 pawn units) or worse (< -0.5 pawn units) for the player to move. All independent variables have been demeaned, allowing main effects to be interpreted as marginal effects at the mean when interaction terms are present. Standard errors are clustered on the game level. *: $p < 0.1$, **: $p < 0.05$, ***: $p < 0.01$.

Table A17. Results split by Evaluation of player to move - Per Subsample

	Dependent Variable: Best Move (Dummy)					
	Subset:Classical Games					
	Subset Positions:					
	All	Winning	Better	Equal	Worse	Losing
	(1)	(2)	(3)	(4)	(5)	(6)
Decision Time						
Time spent on move (min.)	-0.021***	-0.028***	-0.021***	-0.016***	-0.017***	-0.026***
	(0.0007)	(0.002)	(0.002)	(0.001)	(0.002)	(0.003)
Time Budget						
Remaining time (min.)	0.0006***	0.0002	0.002***	0.0002	0.002**	0.003***
	(0.0002)	(0.0008)	(0.0006)	(0.0003)	(0.0007)	(0.001)
Complexity						
N Mega-nodes computed (log)	-0.034***	-0.065***	-0.119***	0.006	-0.115***	-0.056***
	(0.004)	(0.018)	(0.015)	(0.005)	(0.015)	(0.012)
Evaluation Gap						
Distance second best move (log)	0.239***	0.157***	0.284***	0.299***	0.305***	0.145***
	(0.007)	(0.015)	(0.017)	(0.010)	(0.016)	(0.021)
Interactions						
Time spent x Remaining time	0.0003***	0.0003***	0.0003***	0.0003***	0.0002***	0.0005***
	(0.00002)	(0.00009)	(0.00004)	(0.00002)	(0.00005)	(0.0001)
Time spent x N Mega-nodes	-0.004***	0.006	0.0006	-0.005***	-0.002	-0.001
	(0.0008)	(0.005)	(0.003)	(0.001)	(0.004)	(0.004)
Time spent x Dist. second best	0.021***	0.017***	0.020***	0.033***	0.031***	0.008
	(0.002)	(0.005)	(0.004)	(0.003)	(0.005)	(0.008)
Move Observations	80633	7972	10696	45100	9465	7365
Game Observations	1497	738	1127	1370	1050	696
Player-Game Fixed Effects	Yes	Yes	Yes	Yes	Yes	Yes
Control Move Number	Yes	Yes	Yes	Yes	Yes	Yes

Note: Analysis by subset of data whether position of player to move evaluated as winning (better than +2.0 pawn units), better (between +2.0 and +0.5 pawn units), equal (between +0.5 and -0.5 pawn units), worse (between -0.5 and -2.0 pawn units) or losing (worse than -2.0 pawn units) by the Stockfish chess engine. All independent variables have been demeaned, allowing main effects to be interpreted as marginal effects at the mean when interaction terms are present. Standard errors are clustered at the game level, and significance levels are indicated as follows: *: $p < 0.1$, **: $p < 0.05$, ***: $p < 0.01$.

DRAFT

Table A18. Determinants of Decision Quality - Elo Interactions

	Dependent Variable: Best Move (Dummy)		
		Subset:	
	Classical	Rapid	Blitz
	(1)	(2)	(3)
Decision Time			
Time spent on move (min.)	-0.018***	-0.108***	-0.551***
	(0.0005)	(0.003)	(0.018)
Time Budget			
Remaining time (min.)	0.0008***	0.006***	0.022***
	(0.0002)	(0.002)	(0.008)
Complexity			
N Mega-nodes computed	-0.028***	-0.052***	-0.067***
	(0.004)	(0.005)	(0.004)
Evaluation Gap			
Distance second best move (log)	0.198***	0.175***	0.152***
	(0.004)	(0.005)	(0.005)
Interactions			
Elo player x Time spent	0.0003	-0.006	0.007
	(0.0006)	(0.004)	(0.018)
Elo player x Remaining time	-0.00006	-0.004***	-0.006
	(0.0001)	(0.0009)	(0.004)
Elo player x N Mega-nodes	0.011**	0.006	0.001
	(0.005)	(0.005)	(0.004)
Elo player x Dist. second best	0.010*	0.005	0.0007
	(0.005)	(0.005)	(0.005)
Move Observations	80633	62825	73945
Game Observations	1497	1007	1181
Player-Game Fixed Effects	Yes	Yes	Yes
Control Move Number	Yes	Yes	Yes
Control Evaluation Position	Yes	Yes	Yes

Note:OLS estimates. All variables have been demeaned. The variable `Elo player` has been divided by 100. The evaluation of the current position is controlled for using two dummy variables indicating whether the current position is evaluated as better (> 0.5 pawn units) or worse (< -0.5 pawn units) for the player to move. All independent variables have been demeaned, allowing main effects to be interpreted as marginal effects at the mean when interaction terms are present. Standard errors are clustered on the game level. *: $p < 0.1$, **: $p < 0.05$, ***: $p < 0.01$.

DRAFT
